# Influence of Contact Lens Parameters on Cornea: Biomechanical Analysis

**DOI:** 10.3390/bioengineering11100966

**Published:** 2024-09-27

**Authors:** Darshan Ramasubramanian, José Luis Hernández-Verdejo, José Manuel López-Alonso

**Affiliations:** 1 Faculty of Optics and Optometry, Complutense University of Madrid, Arcos de Jalón 118, 28037 Madrid, Spain; darshram@ucm.es (D.R.); jlhernan@ucm.es (J.L.H.-V.); 2 Alain Afflelou Óptico Portugal, Av. António Augusto de Aguiar 11, 1050-016 Lisbon, Portugal

**Keywords:** finite element analysis, ocular biomechanics, contact lens design, wearer comfort

## Abstract

This study presents a finite element analysis to model ocular biomechanics and the interactions between the human eye and contact lenses in the closed-eye condition. The closed-eye state, where the eyelids are fully shut, presents challenges for experimental measurements due to the invasive nature of accessing and analysing the contact lens and corneal interface, making simulation tools valuable for accurate characterisation. The primary objective of this study was to examine how CLs fold and twist and their impact on the cornea when the eye is closed. The secondary aim of this study was to assess how crucial contact lens parameters (Young’s modulus, base curve, and diameter) influence corneal stress distribution and the overall fit of the lens on the eye. The findings show that increasing Young’s modulus significantly reduces corneal stress and promotes uniform stress distribution, making it the most influential factor for wearer comfort and safety. While base curve and diameter variations primarily affect contact area, their impact on stress distribution is minimal. This research provides insights for improving contact lens design and enhancing safety for contact lens wearers.

## 1. Introduction

Contact lenses (CLs) are widely used worldwide, but a significant portion of wearers discontinue use due to discomfort, dropout, or disruptions [1]. Despite advances in CL technology, little progress has been made in addressing this issue. Discomfort can arise from a range of factors, including CL material, incorrect fitting, inadequate care practices, and tear film instability. Additionally, factors such as dry eye syndrome [2], ocular redness, handling problems, and environmental influences such as age, gender, allergies, psychological factors, and medication use play a pivotal role in CL discontinuation [3]. Addressing these contributors is crucial for enhancing the CL wearer experience.

The human eye functions as a complex tribological system characterised by the interaction of a sliding surface: the contact of the eyelid with the cornea and sclera, facilitated by the lubricating properties of the tear film [4]. The tear film, with its multifaceted roles in visual optics, plays a vital role in maintaining ocular health and clarity. Insufficient tear production may lead to dry eyes and may result in corneal abrasions [5,6,7]. When CLs are introduced, these ocular surfaces become divided into two distinct regions. The first exists between the cornea and the back surface of the CL, while the second comprises the front surface of the cornea and the interacting eyelid. The force responsible for preventing relative motion between these surfaces during sliding interactions is referred to as friction [8,9]. This friction is quantified by the coefficient of friction, affecting CL behaviour.

Eyelid–CL interaction and tear surface tension influence CL behaviour [10]. When CLs are introduced, they can alter the dimensions of the eye lens, subsequently affecting its optical power, as previously reported [11,12,13]. These changes can lead to frictional damage on the ocular surface, resulting in various symptoms such as ocular discomfort, dryness, a sensation of foreign bodies, and blurred vision. One of the primary contributors to ocular discomfort among CL wearers is thought to be the combination of increased contact pressure, tear film rupture, and the resulting frictional shear stress [14,15].

There have been many CL designs that have been developed in the past, tailored to different types of CLs [16]. For CLs, various methods have been employed for their design, including base curve selection based on effective power changes for spherical and toric lenses [11,12,13]. The design process assesses the effects of soft CL fitting on the eye, as the interaction between the CL surfaces, tear film, and the eye leads to alterations in optical power, prompting adjustments in the shape of the CL [17].

Numerous CL materials have been examined, mainly focusing on Young’s modulus, measuring stiffness under tension or compression [18]. This modulus, defined as the ratio of stress to strain [19], quantifies a material’s ability to stretch and deform. An increase in Young’s modulus corresponds to reduced contact area and elevated contact pressure, which, in turn, can lead to corneal damage [15,20]. There is also a mathematical relation between the coefficient of friction and velocity that proves the friction coefficient observes a sharp decline with an increase in the velocity caused by the change in the modulus of elasticity [21]. This study investigates five distinct CL materials to understand their impact on the structural dynamics of the human eye in contact with CLs.

Previous studies have conducted simulations and measurements to assess the friction and potential corneal damage caused by the movement of CLs during blinking [8,15,22,23,24]. However, there has been no focus on the simulations and measurement of corneal damage caused by CLs when the eye is closed. This scenario is significant for CL wearers who may choose to wear lenses while sleeping or during activities like meditation or resting. Experimental verification of CL behaviour when the eye is fully closed is challenging. Therefore, this study focuses on simulations to understand the behaviour of the CL on the eye when it is fully closed for a certain period, providing insights that are otherwise unattainable through direct measurement.

The primary objective of this study was to analyse how CLs fold and twist and their impact on the cornea when the eye is closed. A secondary aim of this study was to identify which CL parameters are most associated with corneal damage for future CL design procedures. As commonly used in the literature, a simplified approach was adopted to simulate a closed eye condition, where the entire CL receives uniform pressure. The 3D modelling of this study was focused on the most critical interaction, namely between the CL and the cornea, which was a crucial objective of this study.

To address this, simulations explore the effects of various CL parameters on the ocular surface, particularly material type and design. This article aimed to investigate parameters that may contribute to frictional damage on the ocular surface caused by CLs, focusing on Young’s modulus of the lens material and the base curve and diameter of the CL design in the context of a fully closed eye. The subsequent section outlines the methodologies employed in the design, material properties, and meshing, as well as the load and boundary conditions applied to the human eye model and the CLs. Section 3 discusses the results, followed by the discussion and conclusion in the subsequent section.

## 2. Materials and Methods

For designing and analysing complex dynamic and structural systems, finite element analysis (FEA), utilising the finite element method (FEM), is the widely adopted method. FEA allows precise geometry definition and facilitates the measurement of subtle geometric changes [25]. This allows the evaluation of a physical system under numerous external pressures, analysing stresses and displacements. In this study, a computational model of the human eye and CLs was developed from scratch using MATLAB software [26]. This model was then integrated into the finite element software FEBio Studio [27] to simulate the movement of the CL over the eye in a closed-eye scenario. The following sections detail the geometry, material properties, meshing, and load and boundary conditions required for the analysis of the FEM model.

### 2.1. Modelling

Eye shape varies among individuals, and, for this study, average ocular parameters obtained through an extensive literature review were adopted. Initially, the eye model was created in a two-dimensional (2D) space using MATLAB [26]. Subsequently, this 2D representation was transformed into a three-dimensional (3D) solid model by revolving the 2D arcs, resulting in 3D hemispherical geometries representing the cornea and sclera. Additionally, rotational symmetry along the optic axis was assumed, leading to the composition of the eye model consisting of both the cornea and sclera. In this study, fundamental parameter values, such as cornea outer radius, sclera outer radius, and CL parameters, were sourced from the established literature [11,12,13,28,29,30]. These values were chosen for their reliability and relevance to ocular biomechanics and CL performance, having been widely validated.

In the computational model of the human eye, special attention was devoted to its intricate geometric structure, focusing on both the cornea and the sclera [28]. Instead of resorting to a simplistic representation that might depict these parts as mere uniform shells, the model in this study captures their complex anatomical nuances. Specifically, the model represents the variable thickness inherent to both the cornea and the sclera [28].

**Figure 1:**
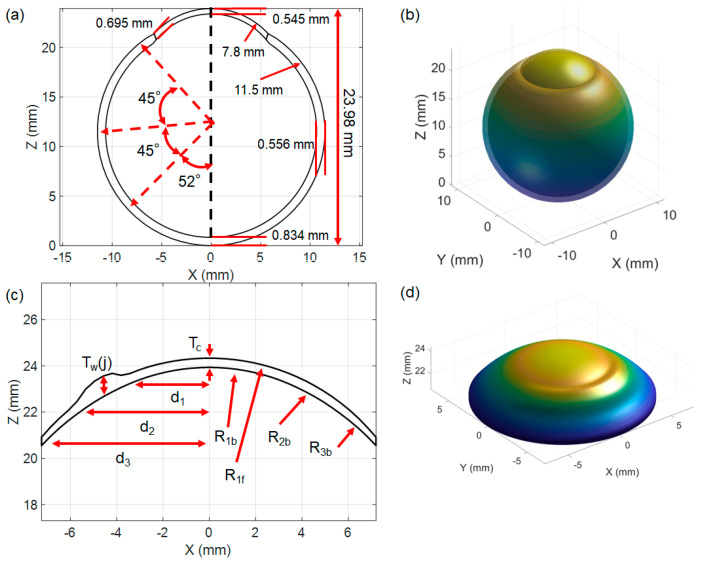
(**a**) Sketch of the 2D model of the sclera and cornea with the optical axis (black dotted line); (**b**) 3D representation of the cornea and sclera; (**c**) front surface (red) and back surface (black) of the CL [11,12,13]; (**d**) 3D visualisation of the CL geometry shown in (**c**). Parameters in the figure are described in the text. The red arrows represent the geometrical parameters and dimensions of the model.

In detailing the parameters of the model, the sclera is characterised by an outer radius of 11.5 mm [28,29]. Similarly, the cornea has an outer radius of 7.8 mm [28,30]. Adjacent to the cornea is the limbus, followed by three distinct segments representing different sections of the sclera. The retina, a vital layer for visual perception, is adjacent to the vitreous chamber. Angular measurements spanning from the central axis to various points on the scleral and retinal structures provide a comprehensive understanding of the internal geometric relationships of the eye. Additionally, specific distances are highlighted [28], such as the 23.93 mm span from the end of the sclera to the cornea, and other thicknesses like 0.695 mm, 0.834 mm, and 0.556 mm at various points. Figure 1a offers a detailed cross-sectional view of a computational human eye model, while Figure 1b provides a 3D visualisation, both displaying its geometric and anatomical complexities.

Previous studies have explored various CL geometries, detailing characteristics such as thickness, base curve, diameter, and material properties [11,12,13,31,32]. In this approach to designing a tri-curve lens with specific geometrical attributes, a MATLAB R2023b program was employed to create the CL surfaces, necessitating precise elements and nodal definitions. The design process involved separate consideration of the front and back surfaces. The back surface was meticulously crafted to achieve an optimal fit, while the front surface was purposefully shaped to align with the desired optical power. Figure 1c displays the designed back and front surfaces of the CL, while Figure 1d offers a 3D representation of the CL.

The initial design of CLs relies on various parameters, and the design procedure is outlined in the references [11,12,13]. Following the guidelines from these references, the back and front surfaces of the CLs were independently crafted. Specifically, the back surface of the CL was divided into three distinct zones: the optic zone (with a lens diameter of d1 = 8 mm), the transient zone (with a lens diameter of d2 = 11.25 mm), and the peripheral zone (with a lens diameter of d3 = 14.5 mm). The base curve for the back surface was determined as Bc = 8.8 mm. Each zone possessed a distinct radius of curvature: R1b = Bc = 8.8 mm for the optic zone, R2b = Bc + 0.5 mm = 9.3 mm for the transient zone, and R3b = Bc + 1 mm = 9.8 mm for the peripheral zone. These parameters collectively contributed to the calculation of the back surface height (Zb) [11,12,13].

The front surface of the CL was tailored to achieve the desired optical power, with a specific power of −2 Dioptres assigned for this case. Employing the prescribed lens power along with a central thickness (Tc) of 0.4 mm and an edge thickness (Te) of 0.4 mm, the front radius of curvature was calculated (R1f = 9.395 mm). These front surfaces were shaped by a lens shape factor set to 1 and the meridional shaping of the front surface height (Zf) [11,12,13]. A weighting factor (W) was introduced to accommodate the prism ballast in the lens, ensuring proper eye orientation for stability and comfort. The weighting factor is defined as W = 0.2 for 0° ≤ θ ≤ 180°, and W = 1 for θ > 180°. The thickness at the boundary between the transient zone and the peripheral zone (Tw (j)) was adjusted to increase the thickness in the lower part of the lens based on the weighting factor, central thickness (Tc), and meridian angles (j = 0°, 1°, 2°…359°). Precisely, Tw (j) is calculated using the formula Tw (j) = Tc × (1 − W × sin (θj)), as shown in Figure 1c. This approach was obtained from the established literature [11,12,13].

Subsequently, a piecewise cubic interpolation method was applied to both CL surfaces [33]. This ensured that the surfaces remained smooth, preserving the integrity of the designed points. This approach enabled the accurate alignment of the CL, where the back and front surfaces were positioned at 24.006 mm. This specific separation aligns with the anatomical distance from the endpoint of the sclera to the cornea, which measures 23.93 mm. These surfaces were further revolutionised to form a 3D model closely resembling the cornea and sclera. In the latter stages of this study, modifications were made to certain CL parameters, specifically the base curve and diameter, to ascertain their influence on the eye and the consequent effects on output parameters.

Although the cornea and sclera are modelled as axisymmetric, the CL used in this study includes a prism ballast, a critical design feature at the bottom part of the lens, intended to stabilise the lens and prevent unwanted rotation. The prism ballast introduces asymmetry into the lens as it is present in the bottom part of the CL, making it essential to utilise a 3D model to accurately capture its effects on lens behaviour. This study focuses on understanding the mechanical interaction and deformation of the CL, excluding the 3D modelling of the eyelid to allow for a concentrated analysis of lens behaviour. The following section will cover the meshing of these 3D geometries and the tools employed for processing these meshes.

### 2.2. Material Properties

The modelling of the sclera has been extensively studied, with various materials such as Neo-Hookean [28,34,35], rigid [36,37], and Ogden [35,38] being explored. It has been observed that the CL placed over the cornea has minimal impact on the deformation of the sclera. After analysing different material options, it became evident that the scleral tissue exhibits small deformations. Considering the simplicity and appropriateness of the model, the Neo-Hookean material model was selected for the sclera [35], and the description of the strain energy function is given in Equation (1), implemented in FEBio [27,39].

(1) $$\Psi (Pa)=\frac{\mu }{2}\left({\bar{I}}_{1}-3\right)-\mu \ln J+\frac{\lambda }{2}{\left(\ln J\right)}^{2},$$

where Ī_1_ is the first invariant of the Cauchy–Green deformation tensor (dimensionless unit), J is the determinant of the deformation gradient tensor (dimensionless unit), and μ (in Pa) and λ (in Pa) are the Lamé parameters related to Young’s modulus (E, in Pa) and Poisson’s ratio (ν, dimensionless) as depicted in Equation (2) [39]:

(2) $$\mu (Pa)=\frac{E}{2\left(1+\nu \right)},\;\lambda \;(Pa)=\frac{\nu E}{\left(1+\nu \right)\left(1-2\nu \right)},$$

The Lamé coefficient (μ) of 0.226 MPa and an average Poisson’s ratio (ν) of 0.47 for the sclera [35,40] were utilised in Equation (2) to calculate the Elastic modulus, resulting in a value of 0.6644 MPa.

In contrast to the simplified model of the sclera, modelling the cornea in the human eyeball presents unique challenges. Numerous mechanical models have been proposed in previous research, including Neo-Hookean [28,35,41,42,43], Mooney–Rivlin [43,44,45], Ogden [35,46], and an anisotropic, hyperelastic large-deformation constitutive model [43,44,47,48]. The cornea exhibits deformability under pressure due to its high water content, allowing it to be modelled as nearly incompressible [44]. Additionally, like other biological materials, the cornea shows highly nonlinear behaviour, and its mechanical properties can vary with factors such as age, hydration level, and health conditions [43]. These characteristics make it essential to consider these complexities when developing accurate models and understanding the biomechanical behaviour of the cornea in different contexts.

Previous research predominantly models the cornea using hyperelastic material models, such as Ogden [35,46] and Mooney–Rivlin [43,44,45], to simulate its mechanical behaviour. However, there is a growing recognition of the need to account for the anisotropic properties of the cornea [43,44,47,48], especially considering its layered structure and the significant role of the stroma in contributing to both transparency and mechanical anisotropy. This approach is vital for accurately modelling the behaviour of the cornea during refractive surgery, where fibre orientation—ranging from horizontal or vertical alignment in the central zone to a more random arrangement in the peripheral areas—underscores the complex structural diversity of the cornea [43,44]. Despite the absence of an anisotropic distributed fibre-reinforced material model in FEBio Studio, alternative material models enable effective simulation. This capability is crucial for achieving the primary objective of this study: using FEM simulators to differentiate between various CLs under certain conditions, highlighting the importance of choosing the right model to accurately represent how the cornea behaves.

In this study, the Mooney–Rivlin material model was chosen for its similarity to the anisotropic distributed fibre-reinforced material model. It effectively captures the volumetric and isotropic aspects of the strain energy function of the cornea, characteristics pertinent to hyperelastic models. It also provides an accurate depiction of the stress–strain relationship of the cornea, especially at lower stress levels, where the response is initially linear for minor deformations and transitions to nonlinear with increased material strain. Such a model is deemed sufficient for representing the corneal behaviour in the context of this study. The finalised model, implemented in FEBio Studio as given in Equation (3), utilises a strain–energy function that integrates both volumetric and isotropic components, thereby providing an accurate representation of the mechanical behaviour of the cornea under the specified conditions.

(3) $$\Psi (Pa)=\Psi_{vol}\left(J\right)+\Psi_{iso}\left(\bar{I_{1}},\bar{I_{2}}\right),\;\Psi (Pa)=\frac{K}{2}{\left(\ln J\right)}^{2}+C_{1}\left(\bar{I_{1}}-3\right)+C_{2}\left(\bar{I_{2}}-3\right)$$

where the volumetric part (Ψ_vol_) is represented by a bulk modulus-like penalty parameter (K), while the isotropic part is described by the Mooney–Rivlin material coefficients C1 and C2. The volumetric component, bulk modulus, and Mooney–Rivlin material coefficients are all expressed in units of Pascals (Pa). At the same time, the invariants (Ī_1_, Ī_2_), and the determinant of the deformation gradient tensor (J) are dimensionless quantities. The constants C1 and C2 were determined based on the study presented in the publication [44], which offers valuable insights and relevant data for the selection of these parameters in the current study. From the previously mentioned reference [44], the mechanical parameters of the underlying mechanical model of the cornea are calculated based on corneal images and their corresponding deformations, no matter whether the model is Neo-Hookean, Mooney–Rivlin, or anisotropic. Despite the higher goodness of fit for the model with anisotropy (as expected), the Mooney–Rivlin model provides a close fit (in the same order of magnitude), making its parameters the choice for introduction in the simulation. The final modelling approach for the cornea is a hyperelastic material represented by a Mooney–Rivlin model, and the material coefficients chosen are C1 = 0.07 mpa and C2 = 0.09 mpa with a bulk modulus K = 7 mpa. The Mooney–Rivlin model, employed in previous studies for experimental measurements on human and porcine corneas, suggests optimal C1 values ranging from 0 to 0.3 mpa and C2 values from approximately −0.06 to 0.3 mpa, aligning with the parameters used in the referenced study [45,46,49].

Focusing on the interaction between the modelled cornea and CLs, this exploration delves into the essential considerations for representing CL behaviour within the computational framework. The Neo-Hookean model is considered suitable for CL simulation as it reduces to a linear elastic model when the strain and rotations are small, aligning with the conditions in this study [11,12,13]. This allows it to replicate the close linear experimental behaviour observed in most materials used in CLs. However, the model also incorporates a slight nonlinearity, making it more adaptable to deviations from perfect elastic behaviour that might be present in certain CL materials. In this model, the strain energy function Ψ of the material is described by Equation (1), which is implemented in the FEM software used (FEBio Studio v2.7.0).

This study examines various material properties of CLs and their effects on the cornea. The properties, including Young’s modulus, base curve, and diameter, are chosen based on those of commercially available CLs, as outlined in Table 1 [32]. The values of the CLs are taken from the published paper by Kim et al. [32]. The Poisson’s ratio and the density of the CLs are assumed constant at 0.49 and 1500 kg/m^3^, respectively. This assumption on density is based on research in tear film dynamics involving CLs [50], due to limited data on these properties for commercial CLs in the existing literature.

This study employed two analytical approaches for examining CLs. The first set maintained a constant base curve and diameter, varying only Young’s modulus to assess its isolated impact on corneal interaction for evaluating how material stiffness influences corneal response independently of CL shape. The second set adjusted Young’s modulus, base curve, and diameter according to specific CL brand specifications, examining their collective effect on the CL–cornea interaction. This comparison aims to differentiate the individual and combined influences of material stiffness and geometric dimensions on CL performance, enhancing our understanding of factors affecting CL comfort and functionality.

### 2.3. Meshing

For computational analysis, meshing represents a pivotal step, entailing the subdivision of complex geometries into more manageable elements. This process unfolded in two distinct phases: pre-processing mesh structures and the subsequent application of the TetGen method.

In the initial phase, MeshLab software v2023.12 [51] emerged as a valuable tool, boasting a versatile array of features tailored to the processing and refinement of mesh structures. This stage focused on critical tasks such as the elimination of duplicate faces and vertices, the consolidation of closely positioned vertices, and the recalibration of vertex normal. Upon meticulous optimisation, the mesh was exported as a pristine model to FEBio Studio v2.7.0 [27], where its surfaces were meticulously verified for accurate representation.

Following the pre-processing phase, the TetGen meshing method came into play to further enhance the refinement of the model. This technique hinged on initial parameter values, including an element size of 1, a quality threshold of 2, and the adoption of tet4 as the element type. These parameters played a vital role in the meticulous meshing of all three geometries, ensuring the robustness of subsequent computational analyses.

After the initial meshing with the prescribed parameters, a mesh convergence study was meticulously conducted to evaluate the Z displacement of the CL at the central node and the computational time, utilising the FEBio software. The initial modelling phase featured the CL1 type parameters from Table 1 and 4-node tetrahedral elements, aligning with the previously mentioned initial mesh parameter values. At this initial phase, the mesh is rudimentary, consisting of 870 nodes for the CL.

**Figure 2:**
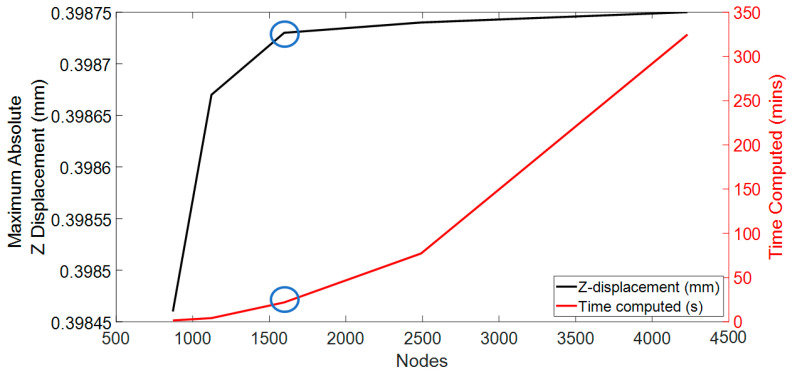
Mesh convergence study with selected configuration represented by the blue circle (Nodes shown in the x-axis are for the CL model).

The methodology of this mesh convergence study was executed in a sequential and methodical fashion, characterised by gradual enhancements of the granularity of the mesh. The initial coarse mesh configuration in the study consists of 30 discrete points, delineating the spatial domain of the geometry created using MATLAB. This initial setup served as a baseline for subsequent iterations, which saw a consistent increase in the number of points to refine the mesh. The iterative process culminated in the development of the finest mesh, delineated by 45 discrete points. The detailed results of this meticulous convergence analysis are presented in Figure 2. This figure provides a visual representation of the convergence behaviour, elucidating the relationship between mesh fineness, Z-axis displacement accuracy, and the overall computational efficiency of the simulation.

Additionally, the computational efficiency of each mesh was considered, particularly the time required to simulate the different meshes. The computer processor utilised for this study was the 11th Gen Intel (R) Core (TM) i5-1135G7 running at 2.40 GHz with a maximum clock of 2.42 GHz. The system was equipped with 8 GB RAM and operated on a 64-bit Windows 11 operating system. The finest mesh required almost 1.5 days for simulation, whereas the coarse mesh took approximately 2 min. Striking a balance between accuracy and computational efficiency was a critical consideration during the evaluation process of the mesh numbers. The mesh numbers from configuration 3 were selected based on these factors to achieve a suitable compromise between accuracy and reasonable computational time.

According to convergence and time computation, mesh configuration 3 was deemed the optimal choice, with a computational time of approximately 15 min. For the sclera and cornea, this configuration featured a 4-node tetrahedral element type, encompassing 8762 nodes and 2963 nodes, respectively. The CL was designed with the same 4-node linear tetrahedral element type with 1597 nodes. These numbers closely align with the findings reported in previous publications [11,12,13,52,53].

### 2.4. Load and Boundary Conditions

The load conditions include three uniform pressures: intraocular pressure (IOP), pressure induced by the surface tension generated by the tears, and eyelid pressure during closed-eye conditions. An IOP of 15 mmHg (≈2 kPa) was applied to the inner surface and connecting surface of the cornea [54].

A pressure induced by the surface tension of the tear film is applied to the back surface of the CL and the outer surface of the cornea. This parameter is closely related to the post-lens tear film [50], a thin layer of fluid situated between the CL and the cornea. This tear film reaches a minimal thickness when the eye is closed. At this point, the pressure exerted primarily originates from the surface tension of the tear fluid, allowing for a near-uniform application of pressure across both the back surface of the CL and the outer surface of the cornea. The average value of the tear film surface tension has been derived from experimental data provided in references [10,11,12,13,55]. Given that the post-lens tear film is in contact with both the CL and cornea, this pressure related to surface tension is uniformly applied to both surfaces. Therefore, the value of 43.6 mPa, used in previous studies [11,12,13,55], was adopted as an approximate value in this study.

**Figure 3:**
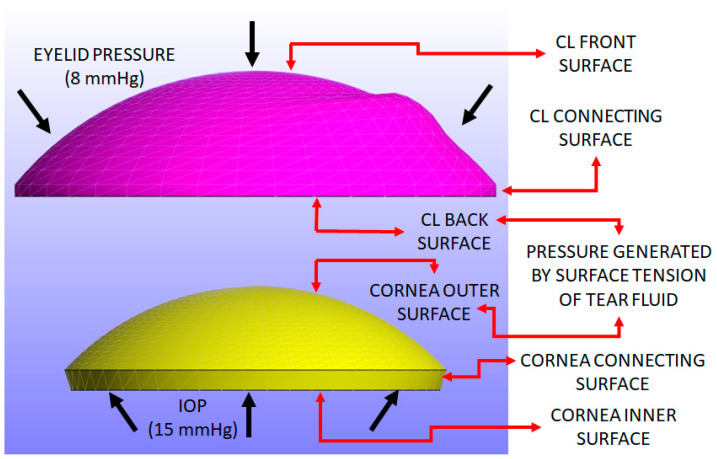
Pressure loads applied on the eye and the CL (represented as black arrows): intraocular pressure (IOP) of 15 mmHg on the inner surfaces of the cornea [54]; tears surface tension (43.6 mPa) applied on the outer surface of the cornea and the back surface of the CL [10,11,12,13,55,57,58]; an eyelid pressure of 8 mmHg was applied on the front surface of the CL [11,12,13,55,56]. The inner, outer, and connecting surfaces of both the cornea and sclera are illustrated (marked by red arrows).

A closed eyelid pressure of 8 mmHg (approximately 1.07 kPa) is applied to the front surface of the CL, as reported in the previous literature [56]. This pressure is applied over a 0.3-s interval, following a parabolic load curve where the maximum pressure of 8 mmHg occurs at around 0.15 s and then returns to zero at 0.3 s. This model simulates the pressure exerted by actions such as pressing the eye with a finger, with the eyelid pressure value obtained from established studies [11,12,13,55,56]. When the eye is fully closed, this pressure becomes more evenly distributed across the corneal surface. A uniform pressure assumption, commonly used in prior research [11,12,13,23,55,56], was adopted to simplify the finite element analysis. The focus remains on understanding how CL parameters, such as material properties, thickness, and curvature, impact the cornea. Figure 3 shows the pressure distribution applied to the CL and cornea, resulting in Z-displacement.

In this study, the inner surface of the cornea is defined as the bottom surface where the IOP is applied. The outer surface is the top surface exposed to the CL, with the connecting surface between these two. For the sclera, the inner surface corresponds to the internal surface facing the intraocular structures, while the outer surface is the external layer in contact with the eye socket, and the connecting surface is the interface between these two layers. For the CL, the back surface is the side in contact with the cornea, the front surface faces the eyelid, and the connecting surface transitions between these two areas. Figure 3 illustrates the surfaces of both the cornea and the CL where the load is applied.

The sclera and cornea were each subjected to Dirichlet boundary conditions, constraining displacement and rotation in all directions on their inner surfaces and connecting surfaces, allowing us to observe the effects on their outer surfaces. In contrast, under a closed-eye assumption for the CL, the model posits that the CL moves freely within the eye, reflecting no constraints on its movement when the eye is shut. Additionally, it is noteworthy that reported coefficients of friction, typically on the order of 0.01 [11,12,13], exhibit variations among different authors due to their origins from non-in vivo measurements and a lack of standardised protocols.

The upcoming section presents the results of the study conducted in a closed-eye scenario, focusing on various key output parameters that can help identify the discomfort associated with wearing CLs.

## 3. Results

This section evaluates the results obtained from the FEM model, focusing on key output parameters such as the displacement of the CL, contact pressure, and contact area on the cornea. To investigate the relationship between CL parameters and their effects on the cornea, six different CLs were considered, each characterised by variations in material and design parameters (see Table 1).

**Figure 4:**
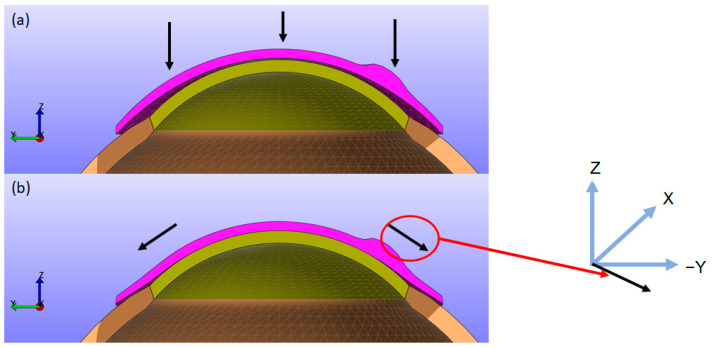
(**a**) FEM model showing the eye (cornea in yellow and sclera in brown), CL (in pink), and eyelid pressure pre-fitting; (**b**) post-fitting model with arrows indicating the radial spread of the CL. The arrows represent the CL direction, movement over the cornea under pressure and the 3D axis.

As depicted in Figure 4, the CL moves closer to the cornea along the Z direction while simultaneously folding and twisting toward the cornea in the X (left-right) and Y (up-down) directions. This results in displacements in the X, Y, and Z directions. Upon contact with the cornea, the lens can cause small displacements on the corneal surface. To provide a clearer understanding of this biomechanical behaviour, figures showing the X, Y, and Z displacements of the CL from a top view, along with sectional views similar to Figure 4, are included for all simulations. Additionally, to improve the clarity of the results, the displacements in the X, Y, and Z directions, as well as the displacement in the XY plane and the total magnitude, for the back surface of the CL and the outer surface of the cornea are provided in the first section of the Supplementary Material.

Displacements play a crucial role in understanding the mechanical behaviour and interaction of CL with the cornea [59]. Displacement data in the X, Y, and Z directions provide insights into how the lens deforms, fits, and moves relative to the corneal surface. Contact pressure is another critical factor when a CL is placed over the cornea, as it influences both the physiological response of the cornea and the overall comfort and effectiveness of the CL. However, in FEBio, spotty contact pressure distributions are inevitable for two reasons: (1) contact is enforced at discrete points rather than over a continuous surface, and (2) planar elements are used to model curved surfaces. These factors often lead to scattered, noisy disturbances in the contact pressure distribution, particularly on curved surfaces. While other software like Abaqus employs built-in smoothing techniques to mitigate such noise, the ease of use and open-source accessibility of FEBio motivated the exploration of alternative methods for evaluating contact interactions.

The 3-principal stress (σ3) was proposed as an alternative to contact pressure for assessing the impact of the CL on the cornea. Since the simulations in this study are conducted under closed-eye conditions, where shear stresses are minimal or negligible, the three principal stresses (σ1, σ2, σ3) become critical for understanding the internal stress state within the corneal tissue. Principal stresses are normal stresses at a point within a material, aligned along specific directions [59]. Among these, σ3 is particularly significant for evaluating CL fit, comfort, and corneal health. Analysing σ3 provides a reliable representation of the compressive forces exerted by the lens, which are crucial to understanding its interaction with the cornea.

The choice of σ3 stress is also crucial since the expected deformations and pressures can occur mainly in the Z direction due to the external pressure on the lens. However, the maximum shear stress was also calculated to evaluate the total deformation. In FEBio, the maximum shear stress is defined using all three principal stresses, as shown in Equation (4). This formula calculates the shear stress between each pair of principal stresses and selects the highest value, ensuring that the most critical shear stress condition is captured.

(4) $$\Xi \left(F\right)=\max\left(\frac{\left|\sigma_{1}-\sigma_{2}\right|}{2},\frac{\left|\sigma_{2}-\sigma_{3}\right|}{2},\frac{\left|\sigma_{3}-\sigma_{1}\right|}{2}\right)$$

where σ1, σ2, σ3 are the three principal stresses. Then, a high correlation between the values of σ3 and $\Xi \left(F\right)$ means that σ3 captures the main deformation direction with accuracy, while a low correlation means that the maximum stress direction is not in the Z direction. These comparisons have been provided for the front and back surfaces of the contact lens and the outer surface of the cornea for all the simulations. Most of the results are included in Section S2 of the Supplementary Materials.

The contact area is determined using the σ3 stress map generated by the FEBio Studio software. Specifically, regions of the corneal surface that encounter the CL are identified and marked (shown in blue in the σ3 stress map). The contact area is then computed for each identified region, considering the specific surface areas described by the mesh. The subsequent subsections examine the influence of each CL parameter on the cornea and sclera. This study employed a time interval corresponding to the standard blink duration of 0.3 s, emphasising the time point t = 0.15 s when maximum eyelid pressure is applied.

### 3.1. Influence of Young’s Modulus

**Figure 5:**
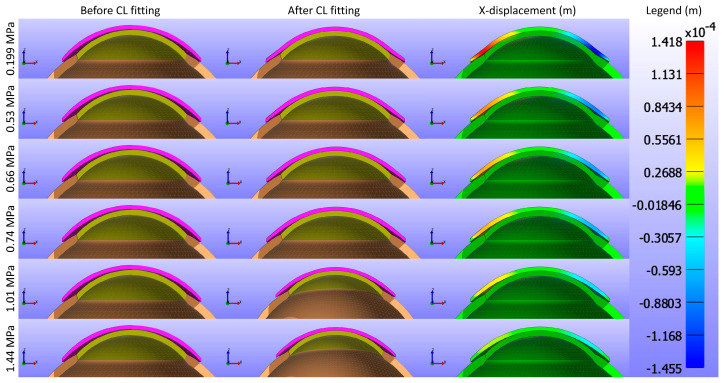
Cross-sectional deformations of the model, including the cornea, sclera, and CL, with variations in Young’s modulus along the X direction. Each row corresponds to different Young’s modulus values. The first two columns show the cross-section view of the overall model before and after fitting. Column 3 represents the X-displacement (m) with a constant legend (in m).

**Figure 6:**
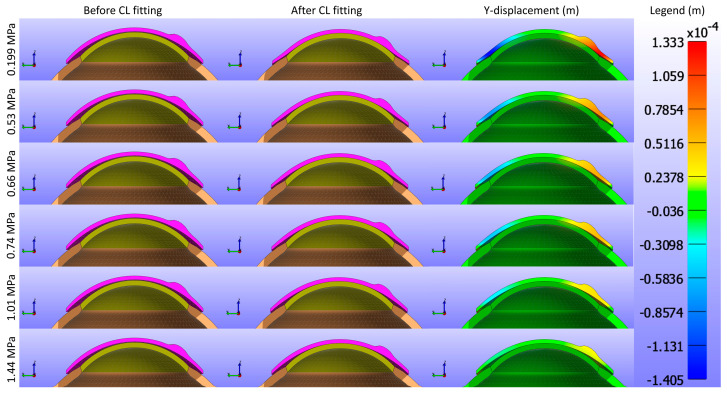
Cross-sectional deformations of the model, including the cornea, sclera, and CL, with variations in Young’s modulus along the Y direction. Each row corresponds to different Young’s modulus values. The first two columns show the cross-section view of the overall model before and after fitting. Column 3 represents the Y-displacement (m) with a constant legend (in m).

This study commenced by evaluating the impact of Young’s modulus of the CL on the cornea while maintaining a constant base curve and diameter for the CL1 type listed in Table 1. Figure 5 and Figure 6 present cross-sectional views of the model deformations, including the cornea, sclera, and CL, showing the effects of varying Young’s modulus in the X and Y directions. Each row corresponds to different Young’s modulus values. The first two columns in Figure 5 and Figure 6 show the cross-section view of the overall model before and after fitting. Column 3 in Figure 5 represents the X-displacement (m) with a constant legend (in m), while column 3 in Figure 6 represents the Y-displacement (m) with a constant legend (in m). The results indicate that changes significantly influence the deformations in Young’s modulus. As expected from structural mechanics principles, materials with higher Young’s modulus values exhibit more minor deformations, as stiffer materials tend to deform less under applied forces.

**Figure 7:**
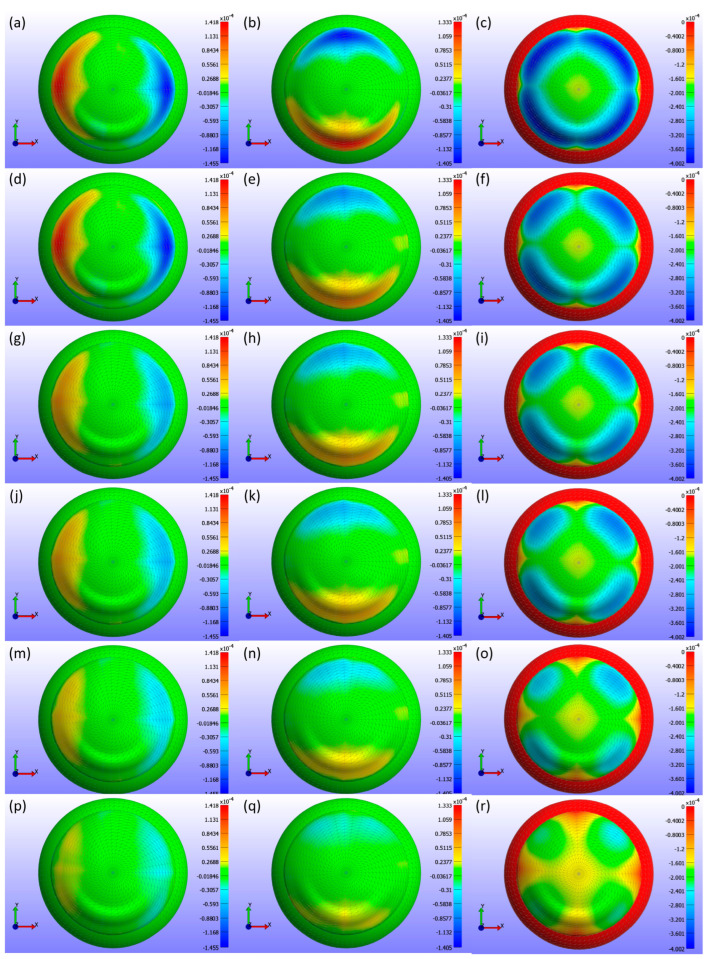
Displacement maps of the CL over the cornea for different Young’s modulus (E) values with a constant legend. The first column (**a**,**d**,**g**,**j**,**m**,**p**) displays the X-displacement (m), the second column (**b**,**e**,**h**,**k**,**n**,**q**) shows the Y-displacement (m), and the third column (**c**,**f**,**i**,**l**,**o**,**r**) presents the Z-displacement (m). The rows correspond to different Young’s modulus values: the first row (**a**–**c**) represents E = 0.199 MPa, the second row (**d**–**f**) E = 0.53 MPa, the third row (**g**–**i**) E = 0.66 MPa, the fourth row (**j**–**l**) E = 0.74 MPa, the fifth row (**m**–**o**) E = 1.01 MPa, and the sixth row (**p**–**r**) E = 1.44 MPa.

Displacement maps generated from FEBio Studio, illustrated in Figure 7, depict the movement of the CL over the cornea for various Young’s modulus values. The first column in Figure 7(a,d,g,j,m,p) displays the X-displacement of the CL over the cornea, the second column in Figure 7(b,e,h,k,n,q) shows the Y-displacement, and the third column in Figure 7(c,f,i,l,o,r) presents the Z-displacement. The rows correspond to different Young’s modulus values: the first row in Figure 7(a,b,c) represents E = 0.199 MPa, the second row in Figure 7(d,e,f) depicts E = 0.53 MPa, the third row in Figure 7(g,h,i) describes E = 0.66 MPa, the fourth row in Figure 7(j,k,l) represents E = 0.74 MPa, the fifth row in Figure 7(m,n,o) depicts E = 1.01 MPa, and the sixth row in Figure 7(p,q,r) displays E = 1.44 MPa. The legend in the displacement map remains constant across all Young’s modulus values to highlight the variations in displacement, with displacements measured in metres.

As shown in Figure 5, Figure 6 and Figure 7, the displacement in all directions—X, Y, and Z—exhibits a notable decrease with increasing Young’s modulus of the CL. This trend indicates that as the material stiffness of the CL increases, its ability to deform under the applied pressures diminishes. Specifically, a higher Young’s modulus results in a stiffer lens, which maintains its shape more effectively against the forces exerted by the eyelid. Consequently, the lens exhibits reduced movement over the corneal surface. The displacement maps in Figure 5 vividly illustrate this phenomenon, where softer lenses (lower Young’s modulus) show greater deformation and movement compared to stiffer lenses.

**Figure 8:**
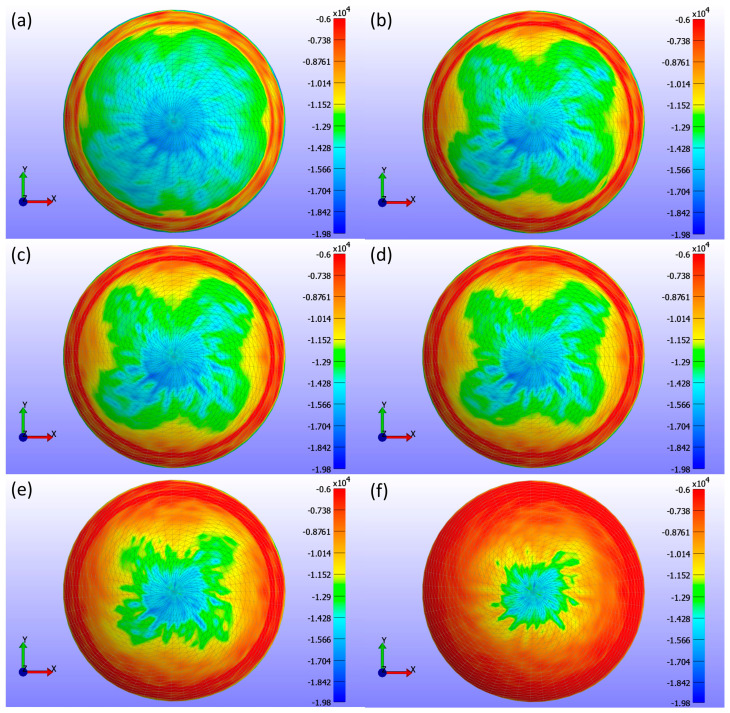
σ3 stress (Pa) maps on the cornea, showing the areas where the CL is placed for various Young’s modulus (E) values with a constant legend. The maps correspond to different Young’s modulus values: (**a**) E = 0.199 MPa, (**b**) E = 0.53 MPa, (**c**) E = 0.66 MPa, (**d**) E = 0.74 MPa, (**e**) E = 1.01 MPa, and (**f**) E = 1.44 MPa.

Figure 8 presents detailed information on the σ3 stress maps (Pa) for various Young’s modulus values. Figure 8a shows E = 0.199 MPa, Figure 8b shows E = 0.53 MPa, Figure 8c shows E = 0.66 MPa, Figure 8d shows E = 0.74 MPa, Figure 8e shows E = 1.01 MPa, and Figure 8f shows E = 1.44 MPa. The figure demonstrates that as Young’s modulus increases, the high-stress area in the centre of the lens decreases, and the stress values become more uniformly distributed across the lens, ranging from 0 to −19.804 kPa. Softer lenses, with lower Young’s modulus, concentrate higher stress in the central region. The contact area, determined by analysing the high σ3 stress values, is outlined in Table 2, which presents contact area measurements for each lens based on Young’s modulus using the σ3 stress map from FEBio Studio. The contact area decreases with increasing Young’s modulus, indicating that stiffer lenses result in less corneal deformation.

### 3.2. Influence of Base Curve

**Figure 9:**
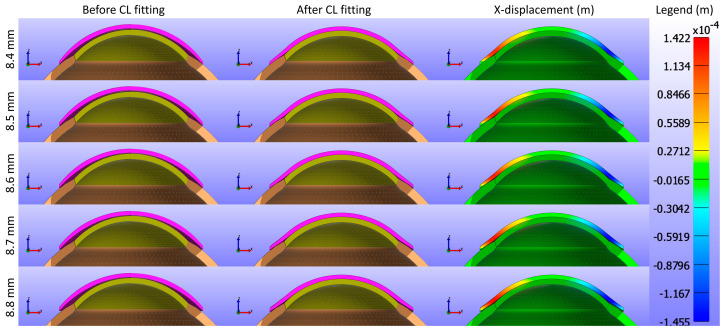
Cross-sectional deformations of the model, including the cornea, sclera, and CL, with variations in the base curve along the X direction. Each row corresponds to different base curve values. The first two columns show the cross-section view of the overall model before and after fitting. Column 3 represents the X-displacement (m) with a constant legend (in m).

**Figure 10:**
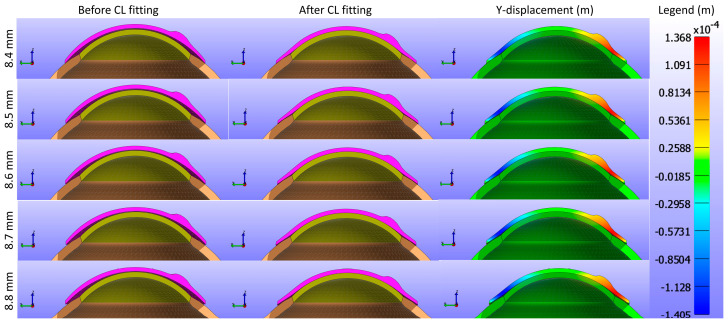
Cross-sectional deformations of the model, including the cornea, sclera, and CL, with variations in the base curve along the Y direction. Each row corresponds to different base curve values. The first two columns show the cross-section view of the overall model before and after fitting. Column 3 represents the Y-displacement (m) with a constant legend (in m).

The study further assessed the impact of the base curve of the CL on the cornea while maintaining a constant Young’s modulus and diameter for the CL1 type listed in Table 1. Figure 9 and Figure 10 present cross-sectional views of the model deformations, including the cornea, sclera, and CL, showing the effects of varying base curves in the X and Y directions. Each row corresponds to different base curve values. The first two columns in Figure 9 and Figure 10 show the cross-section view of the overall model before and after fitting. Column 3 in Figure 9 represents the X-displacement (m) with a constant legend (in m), while column 3 in Figure 10 represents the Y-displacement (m) with a constant legend (in m). The results indicate that variations in the base curve lead to minimal differences in deformation across the corresponding values.

**Figure 11:**
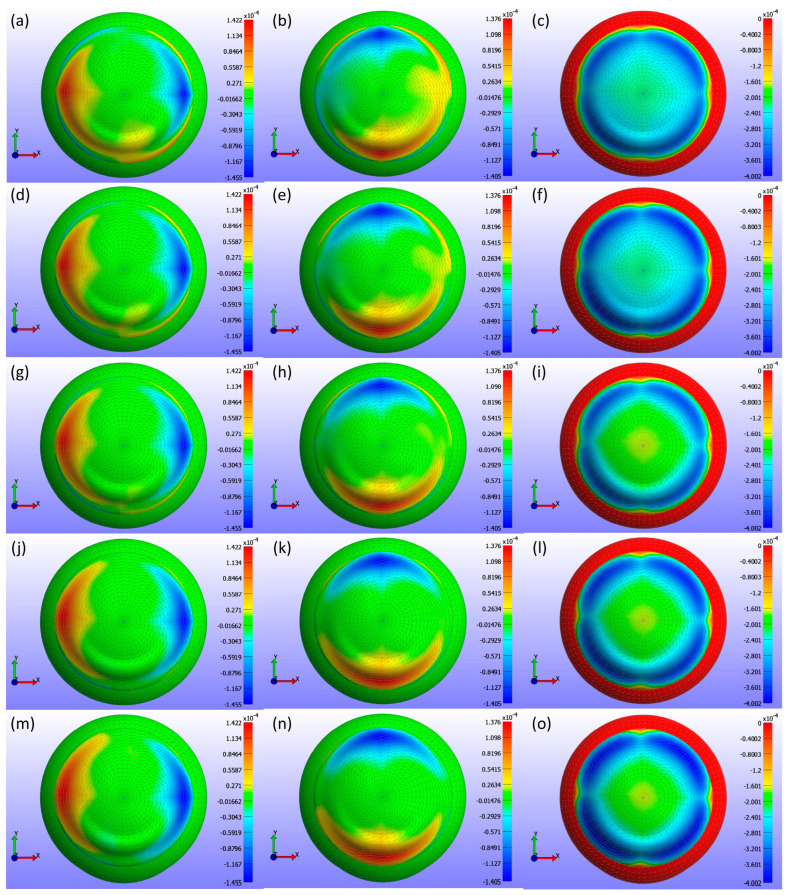
Displacement maps of the CL over the cornea for various base curve (Bc) values with a constant legend. The first column (**a**,**d**,**g**,**j**,**m**) displays the X-displacement (m), the second column (**b**,**e**,**h**,**k**,**n**) shows the Y-displacement (m), and the third column (**c**,**f**,**i**,**l**,**o**) presents the Z-displacement (m). The rows correspond to different base curve values: Bc = 8.4 mm in the first row (**a**–**c**), Bc = 8.5 mm in the second row (**d**–**f**), Bc = 8.6 mm in the third row (**g**–**i**), Bc = 8.7 mm in the fourth row (**j**–**l**), and Bc = 8.8 mm in the fifth row (**m**–**o**).

Displacement maps generated from FEBio Studio, illustrated in Figure 11, depict the movement of the CL over the cornea for various base curve values. The first column in Figure 11(a,d,g,j,m) shows the X-displacement of the CL, the second column in Figure 11(b,e,h,k,n) shows the Y-displacement, and the third column in Figure 11(c,f,i,l,o) shows the Z-displacement. The rows correspond to different base curve values: Bc = 8.4 mm in the first row in Figure 11(a,b,c), Bc = 8.5 mm in the second row in Figure 11(d,e,f), Bc = 8.6 mm in the third row in Figure 11(g,h,i), Bc = 8.7 mm in the fourth row in Figure 11(j,k,l), and Bc = 8.8 mm in the fifth row in Figure 11(m,n,o). The legend in the displacement map remains constant across all base curve values to highlight the variations in displacement, with displacements measured in metres.

As shown in Figure 9, Figure 10 and Figure 11, the X-displacement maps exhibited a consistent deformation pattern across all base curve values, with a slight decrease in displacement magnitude as the base curve increased, indicating reduced X-direction deformation for higher base curves. Similarly, the Y-displacement maps showed a uniform deformation pattern with a gradual reduction in displacement magnitude with increasing base curve values, suggesting decreased Y-direction deformation. In contrast, the Z-displacement maps displayed more pronounced changes, with higher base curves showing significantly lower Z-displacement compared to lower base curves. This indicates that increasing the base curve value significantly reduces Z-direction deformation.

**Figure 12:**
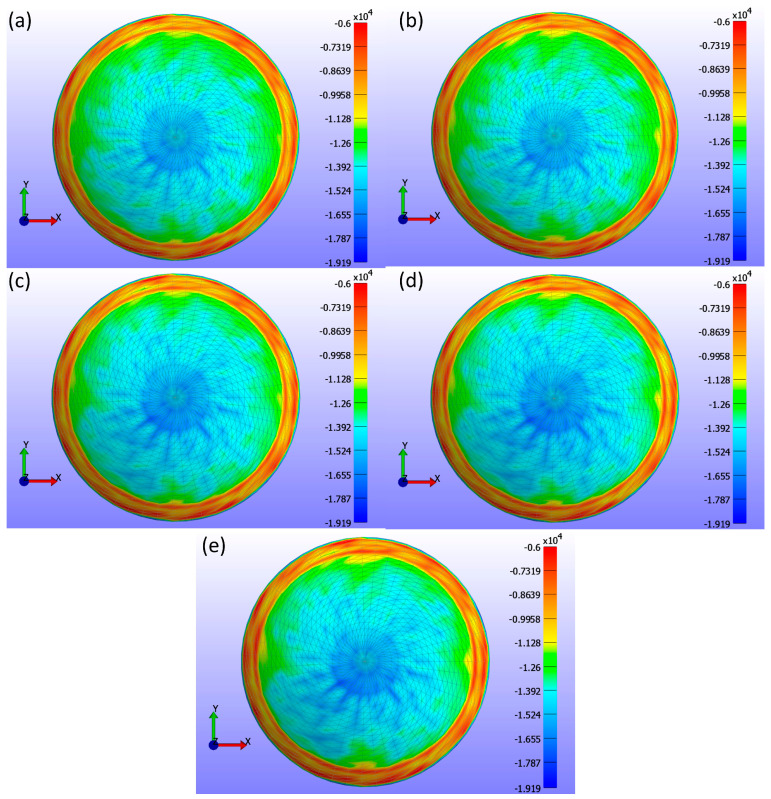
σ3 stress (Pa) maps on the cornea, showing the areas where the CL is placed for various base curve (Bc) values with a constant legend. The maps correspond to different base curve values: (**a**) Bc = 8.4 mm, (**b**) Bc = 8.5 mm, (**c**) Bc = 8.6 mm, (**d**) Bc = 8.7 mm, and (**e**) Bc = 8.8 mm.

The analysis of σ3 stress maps for CLs with varying base curve values is presented in Figure 12. Figure 12a shows a base curve of 8.4 mm, Figure 12b shows 8.5 mm, Figure 12c shows 8.6 mm, Figure 12d shows 8.7 mm, and Figure 12e shows 8.8 mm. The σ3 stress maps indicate only minute changes in stress distribution, which is not uniformly distributed within the range of 0 to −19.193 kPa. These subtle changes are evident in the contact area measurements outlined in Table 3. As the base curve increases, the contact area decreases, demonstrating that the base curve influences corneal stress and CL fitting. This reduction in contact area suggests that lenses with a higher base curve have less surface area in contact with the cornea.

### 3.3. Influence of Diameter

**Figure 13:**
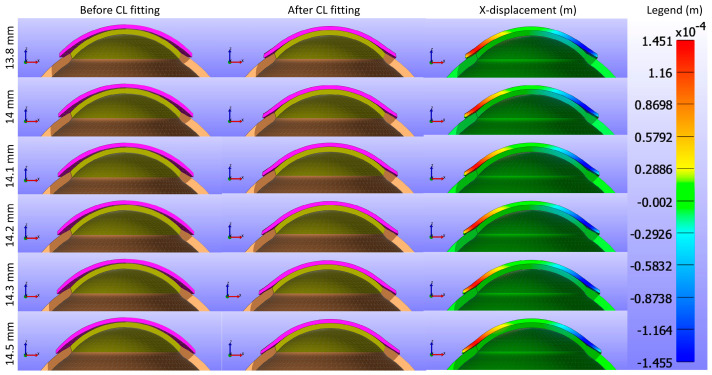
Cross-sectional deformations of the model, including the cornea, sclera, and CL, with variations in CL diameter along the X direction. Each row corresponds to different diameter values. The first two columns show the cross-section view of the overall model before and after fitting. Column 3 represents the X-displacement (m) with a constant legend (in m).

**Figure 14:**
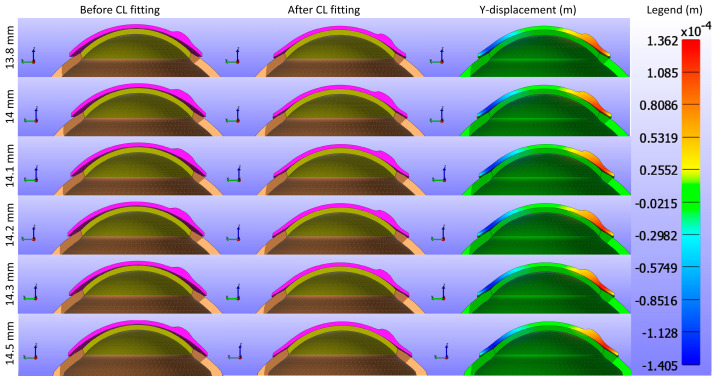
Cross-sectional deformations of the model, including the cornea, sclera, and CL, with variations in CL diameter along the Y direction. Each row corresponds to different diameter values. The first two columns show the cross-section view of the overall model before and after fitting. Column 3 represents the Y-displacement (m) with a constant legend (in m).

In this study, the CL diameter was further investigated to analyse its impact on the cornea while keeping Young’s modulus and base curve constant for the CL1 type listed in Table 1. Figure 13 and Figure 14 present cross-sectional views of the model deformations, including the cornea, sclera, and CL, showing the effects of varying CL diameters in the X and Y directions. Each row corresponds to different diameter values. The first two columns in Figure 13 and Figure 14 show the cross-section view of the overall model before and after fitting. Column 3 in Figure 13 represents the X-displacement (m) with a constant legend (in m), while column 3 in Figure 14 represents the Y-displacement (m) with a constant legend (in m). The results demonstrate that changes in the diameter result in only slight differences in deformation across the respective values.

**Figure 15:**
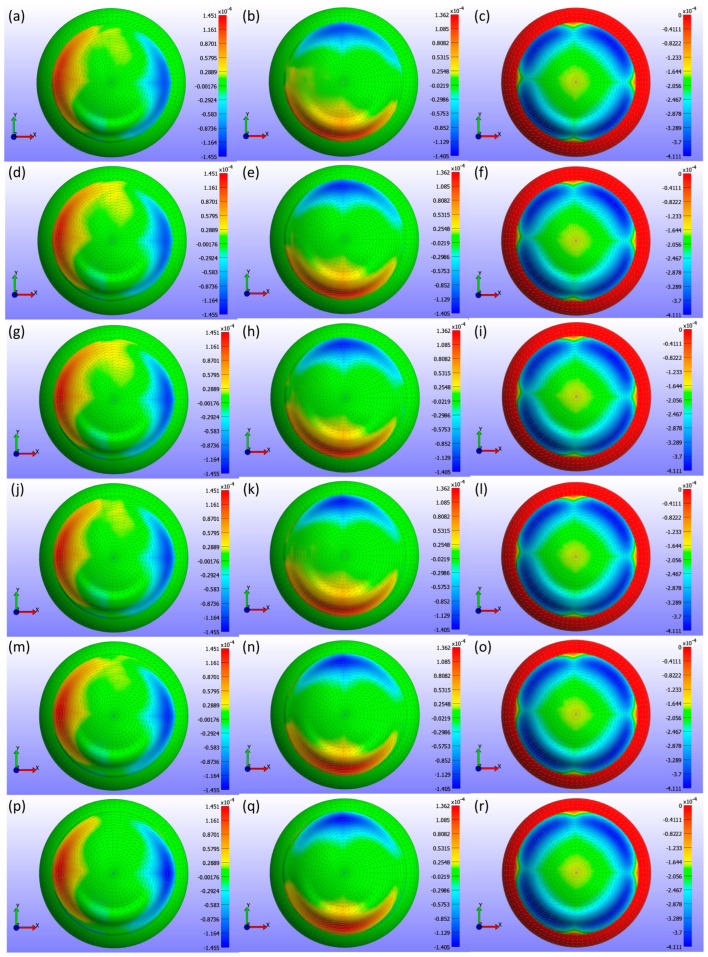
Displacement maps of the CL over the cornea for various diameter (d3) values with a constant legend. The first column (**a**,**d**,**g**,**j**,**m**,**p**) displays the X-displacement (m), the second column (**b**,**e**,**h**,**k**,**n**,**q**) shows the Y-displacement (m), and the third column (**c**,**f**,**i**,**l**,**o**,**r**) presents the Z-displacement (m). The rows correspond to different diameter values: d3 = 13.8 mm in the first row (**a**–**c**), d3 = 14.0 mm in the second row (**d**–**f**), d3 = 14.1 mm in the third row (**g**–**i**), d3 = 14.2 mm in the fourth row (**j**–**l**), d3 = 14.3 mm in the fifth row (**m**–**o**), and d3 = 14.5 mm in the sixth row (**p**–**r**).

Displacement maps generated from FEBio Studio, shown in Figure 15, illustrate the movement of the CL over the cornea for various diameter values. The first column in Figure 15(a,d,g,j,m,p) shows the X-displacement of the CL over the cornea, the second column in Figure 15(b,e,h,k,n,q) shows the Y-displacement, and the third column in Figure 15(c,f,i,l,o,r) presents the Z-displacement. The rows correspond to different diameter values in Figure 15: the first row (a,b,c) represents d3 = 13.8 mm, the second row (d,e,f) d3 = 14.0 mm, the third row (g,h,i) d3 = 14.1 mm, the fourth row (j,k,l) d3 = 14.2 mm, the fifth row (m,n,o) d3 = 14.3 mm, and the sixth row (p,q,r) d3 = 14.5 mm. The legend in the displacement map remains constant across all diameter values to highlight the variations in displacement, with displacements measured in metres.

Figure 15 presents a detailed analysis of the displacement of a CL over the cornea for different lens diameters in three spatial directions (X, Y, Z). The X-displacement and Y-displacement maps indicate that the central region exhibits minimal displacement, with increased variations towards the periphery. Although the displacement patterns change slightly as the lens diameter increases from 13.8 mm to 14.5 mm, these differences are minor. The Z-displacement maps show a very consistent pattern across all diameters, with significant central displacements that decrease towards the periphery, suggesting that Z-displacement is not strongly influenced by changes in lens diameter. The consistent colour patterns across all directions imply a well-centred lens with symmetrical displacement distributions. These observations suggest that while CL diameter can have some effect on the displacement distribution, the overall influence is minimal, particularly for Z-displacement.

**Figure 16:**
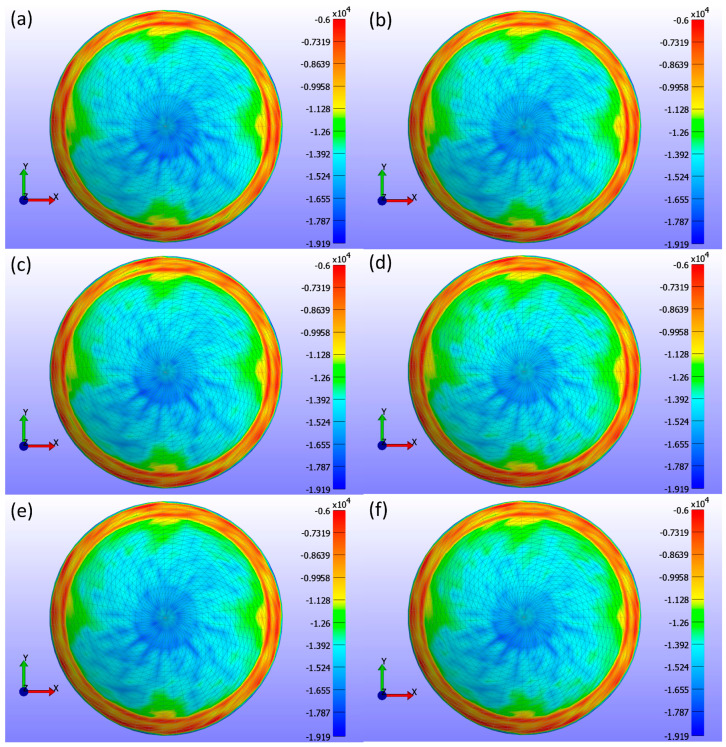
σ3 stress (Pa) maps on the cornea, showing the areas where the CL is placed for various diameter (d3) values with a constant legend. The σ3 stress maps correspond to different diameter values: (**a**) d3 = 13.8 mm, (**b**) d3 = 14.0 mm, (**c**) d3 = 14.1 mm, (**d**) d3 = 14.2 mm, (**e**) d3 = 14.3 mm, and (**f**) d3 = 14.5 mm.

Figure 16 presents a comprehensive analysis of σ3 stress for various CL diameters (d3) as obtained from FEBio Studio. Figure 16a shows a diameter of 13.8 mm, Figure 16b shows 14.0 mm, Figure 16c shows 14.1 mm, Figure 16d shows 14.2 mm, Figure 16e shows 14.3 mm, and Figure 16f shows 14.5 mm. The analysis in the σ3 stress map indicates minor changes in stress distribution across different diameters, with the stress not being uniformly distributed. These changes are even smaller than those observed with variations in the base curve. Table 4 shows the contact area measurements, demonstrating that as the diameter increases, the contact area also increases. This increase is primarily due to the CL design associated with larger diameters, which naturally results in more surface area encountering the cornea.

## 4. Discussion

This study investigates the biomechanical interactions between CLs and the human eye, specifically focusing on the effects of CL mechanical and geometrical properties during eyelid closure. Although substantial literature exists on blinking [8,23], the closed-eye scenario remains underexplored. Experimental verification of the CL behaviour, when the eye is completely closed, is challenging to obtain. To address this gap, CL behaviour during the closed-eye period was analysed using FEA with FEBio software [27] and MATLAB [26], this research aims to fill that gap. The model incorporates the complex anatomical structure of the eye, enhancing realism beyond simplistic representations [11,12,13,28,43]. The findings confirm that the design and negative Z-direction movement of CLs provides a foundation for exploring the impact of various hyperelastic material models on corneal behaviour.

Key output parameters discussed include displacement, σ3 stress, and contact area, which are crucial for assessing corneal health and CL comfort. Displacements in the X, Y, and Z directions provide insights into the mechanical behaviour and interaction of CLs with the cornea [60]. Understanding how the lens deforms and fits on the cornea is essential for optimising lens design. Contact pressure is critical for CL wearers as excessive pressure can lead to discomfort, corneal deformation, and potential damage to the corneal epithelium [8,24]. Due to the spotty nature of contact pressure in FEBio Studio and negligible shear stress from the eye closed, σ3 stress analysis was used as an alternative for contact pressure. σ3 stress is crucial for identifying compressive stresses exerted by the CL on the cornea and provides a reliable representation of these forces [59,61]. The contact area affects how σ3 stress is distributed across the cornea [8,24].

The modelling of the cornea and sclera in this study is axisymmetric, as the primary objective is to understand how the CL behaves under uniform load conditions, such as when the eye is closed. However, accurate 3D modelling was necessary due to the asymmetry of the CL, which contains a prism ballast located at the bottom of the lens. The prism ballast is critical in stabilising the lens and preventing unwanted rotation. The prism ballast is at the bottom part of the lens, and the stress distribution becomes slightly concentrated in that region, demonstrating that the model is non-axisymmetric. This emphasises the importance of the size and location of the prism ballast in evaluating corneal health.

Evaluating the effect of Young’s modulus revealed that increased modulus values lead to reduced radial and axial displacements, indicating decreased corneal deformation. This suggests that stiffer lenses, with higher Young’s modulus, exhibit smaller displacements due to their resistance to deformation. Conversely, softer lenses conform more to the cornea, resulting in larger displacements. Therefore, while softer lenses may offer a better fit, they might be more prone to greater deformation and potential instability.

Regarding contact analysis based on contact area, this study found that the contact area decreases with increasing Young’s modulus. This indicates that stiffer lenses (higher Young’s modulus) have less surface area in contact with the cornea. The σ3 stress maps further illustrate this phenomenon by showing more even and centralised stress distributions for lenses with higher Young’s modulus values. As the stiffness of the lens increases, it conforms less to the irregularities of the corneal surface, leading to a more concentrated stress distribution in the central region. This is critical for understanding how different lens materials affect corneal stress and potential deformation, as well as for optimising lens design to balance comfort and safety for the wearer.

These findings on Young’s modulus have significant implications for corneal health and CL comfort. The association between stiffer silicone hydrogel CLs and increased meibomian gland loss and dry eye symptoms highlights the critical role of lens material properties in maintaining ocular surface health [18]. The observation that stiffer lenses reduce deformation and provide more even stress distributions aligns with the need to balance modulus and oxygen permeability to prevent mechanical irritation and ensure comfort [62]. Additionally, the correlation between Young’s modulus and ocular surface complications, such as conjunctivitis and edge fluting, emphasises the importance of mechanical properties in CL design [63]. The reduced deformation observed with higher modulus lenses suggests that selecting appropriate materials can enhance structural integrity and produce more uniform stress distributions. This insight underscores the necessity for a personalised approach in lens selection, aligning biomechanical compatibility with corneal properties to improve ocular health outcomes. Incorporating these findings into clinical practice can refine CL recommendations and customise materials to enhance comfort and health. Such a tailored approach advocates for innovations in material science that balance wearer comfort and health benefits [63,64], improving the overall efficacy and safety of CL usage in diverse patient populations.

This study further evaluated the impact of the CL base curve on the cornea while maintaining a constant Young’s modulus and diameter. Displacement maps from FEBio Studio illustrate CL movement over the cornea for various base curve values (Bc = 8.4 mm to Bc = 8.8 mm). The X- and Y-displacement maps displayed consistent deformation patterns with a slight decrease in displacement magnitude as the base curve increased, indicating reduced deformation in these directions for higher base curves. The Z-displacement maps showed more pronounced changes, with higher base curves resulting in significantly lower Z-displacement, indicating a substantial reduction in Z-direction deformation.

The analysis of σ3 stress maps for CLs with varying base curves revealed significant variations in stress distributions and contact area. The σ3 stress maps indicated a small increment in the maximum stress value with increasing base curves; lower base curves exhibited lower σ3 stress values, while higher base curves presented higher maximum absolute σ3 stress values. This suggests reduced stress concentrations with lower base curves. Additionally, as the base curve increased, the contact area on the cornea decreased. This decrease in contact area is important because it influences the fitting of the CL over the corneal surface, ensuring that the lens conforms well to the unique shape of the eye. This personalisation helps provide a better fit, reduces the risk of discomfort and potential corneal damage, and improves overall visual performance.

This investigation into base curve variations underscores the nuanced relationship with CL fit and comfort, revealing moderate correlations with contact pressure and area that, while not statistically significant, illuminate the complex challenge of achieving an optimal lens fit [65]. Such findings echo the limitations of traditional fitting methods, where mathematical modelling has proven beneficial in adjusting base curve and diameter to enhance fit success, as demonstrated by high success rates in specific lens designs [66]. Moreover, this study corroborates the existing literature on the inadequacy of central corneal curvature measurements in predicting the optimal base curve [65], highlighting a sophisticated interplay of factors like Young’s modulus in lens selection [63]. Despite the lack of statistical significance, the research supports the subjective importance of comfort and fit, advocating for a personalised approach to lens fitting that transcends conventional metrics and embraces the complexity of individual corneal topographies and mechanical properties for improved wearer satisfaction [67,68].

The third section of the results focused on the CL diameter, evaluating its impact on the cornea and lens while maintaining a constant Young’s modulus and base curve. Displacement maps generated from FEBio Studio illustrate the movement of the CL over the cornea for various diameter values (13.8 mm to 14.5 mm). The X-displacement and Y-displacement maps show only minor changes with increasing CL diameter. The Z-displacement maps reveal consistent central displacements across all diameters, suggesting that Z-displacement is not strongly influenced by changes in lens diameter. These patterns imply a well-centred lens with symmetrical displacement distributions, indicating that CL diameter has minimal impact on overall displacement distribution, particularly for Z-displacement.

A comprehensive analysis of σ3 stress for various CL diameters indicates a minor increase in peak stresses as the diameter increases. This trend suggests that while larger lenses exert slightly higher stresses as diameter increases, the changes are not substantial enough to cause significant concern. Additionally, the contact area expands with larger diameters due to the increase in the overall surface area of the CL. This enhanced contact area improves the lens fit across the corneal surface, thereby enhancing comfort. These findings suggest that while changes in CL diameter have a minor impact on stress distribution, the increased contact area associated with larger diameters can significantly improve lens comfort and fit.

These results align with previous studies that explored the relationship between base curve, diameter, and lens fit using mathematical models. Specific combinations of the base curve and diameter significantly influence lens tightness and corneal overlap, correlating with the finding that CL diameter affects pressure distribution and contact area [66]. Furthermore, research on large-diameter multifocal CLs in presbyopic adults with dry eye syndromes found that larger diameters can positively influence visual function despite minor impacts on the ocular surface [69]. This supports the conclusion that larger CL diameters can enhance comfort and lens fit without significantly affecting corneal displacement patterns. Although CL diameter significantly influences tear film dynamics [70], it does not greatly affect corneal movement because the CL and the eye usually operate under an elastohydrodynamic lubrication regime rather than a boundary lubrication regime [22].

When comparing the simulation results for the CL parameters—Young’s modulus, base curve, and diameter—about displacements, Young’s modulus is found to exert the most significant influence on the folding and twisting of the CL in the X-Y direction. In contrast, the base curve and diameter have minimal impact. This conclusion is supported by Pearson correlation analysis between the CL parameters and the maximum displacements in the X, Y, and Z directions, as shown in Table 5, with a 95% confidence interval. The maximum value of Z-displacement is represented as a negative value as the CL moves in the negative Z-direction. For the X and Y displacements, the absolute values are initially calculated to determine the maximum displacement, with the sign subsequently adjusted based on the direction. If the maximum displacement occurs in the negative direction, the value is reported as the negative of the maximum absolute displacement. The analysis reveals a statistically significant (*p* < 0.05) positive correlation between Young’s modulus and maximum displacements. In contrast, the base curve and diameter show no statistically significant correlation with displacement, except for a statistically significant negative correlation between diameter and the maximum Y displacement.

Furthermore, a Pearson correlation analysis between the CL parameters and the minimum σ3 stress was also performed, revealing statistically significant correlations for all three parameters, as shown in Table 5. The minimum stress values were considered due to the negative direction of the stress on the cornea. Among the parameters, the base curve exhibited the strongest negative correlation, followed by Young’s modulus and diameter, in this order. In terms of contact area, all three parameters showed substantial and statistically significant relationships, as detailed in Table 5. Young’s modulus and base curve were negatively correlated with contact area, while diameter showed a positive correlation. Overall, Young’s modulus plays a crucial role in influencing corneal health and wearer comfort, with the base curve and diameter having a lesser but still significant impact.

These results suggest that the deformation of the CL is influenced by the continuous pressure exerted by the eye, a factor that could affect when considering the spatiotemporal dynamics of the blinking. The potential impact of this CL deformation on the optical characteristics warrants further investigation, which could be explored using advanced simulation tools [11,12,13]. Additionally, the presence of the prism ballast used for CL stabilisation introduces differential effects on the fold and twist of the CL in the horizontal and vertical directions. The models developed in this study provide a foundation for examining how varying the position and size of these prisms may affect lens deformation. The use of accessible tools such as FEBio [27] can facilitate the broader adoption and application of these techniques in laboratory and clinical settings. Future work will extend these findings to more sophisticated multiphysics models, incorporating fluid dynamics and realistic blinking scenarios, to build on the preliminary results presented in this study [71].

Regarding the results given in the Supplementary Material, it can be observed that the correlation between sigma3 and Maximum Shear Stress is high for all surfaces studied and with all simulated parameters. The main deformation of the back surface of the lens occurs in the XY direction and not in Z, where it does occur for the case of the cornea, which is evidenced again by this comparison between the 3-principal stress for these two surfaces.

## 5. Conclusions

This study demonstrates that the mechanical properties and geometrical design of CLs significantly affect corneal health and comfort during the eyelid closure phase, with Young’s modulus, base curve, and lens diameter playing significant roles. Increased Young’s modulus reduces corneal deformation and leads to more even stress distributions, while softer lenses, although offering a better fit, are prone to greater deformation. Variations in the CL base curve influence deformation patterns and pressure distribution, with higher base curves resulting in reduced Z-displacement, more uniform pressure distributions, and lower internal stress magnitudes, thereby improving fit and comfort. Changes in CL diameter, although having a minor impact on overall displacement, significantly affect contact pressure and stress distribution, with larger diameters providing a more even pressure distribution and enhancing overall comfort and fit. These findings highlight the importance of mechanical and geometrical properties in CL design and suggest that CLs should be chosen based on how well they match the corneal characteristics of each individual. This personalised approach can improve eye health and make CL use more effective and safer. Looking ahead, this work recommends an advanced multiphysics approach to incorporate tear film dynamics to better understand the cornea, tear fluid, and CL interactions during eyelid movements. This approach could reveal the complex factors involved in their interactions, leading to an improved CL design and better safety and comfort for users.

## Figures and Tables

**Table 1:** Material properties of the CLs. CL1—reference CL [11,12,13]; CL2—Air Optix Day & Night Aqua CL; CL3—Dailies CL; CL4—Acuvue Oasys CL; CL5—Dailies Total 1 CL; CL6—MyDay CL. Data from Table 1 of Kim et al. [32]. The mean and standard deviation (std) of the parameter ranges are given at the bottom row of this table.

CL	Young’s Modulus (MPa)	Base Curve (mm)	Diameter (mm)
CL1	0.199	8.8	14.5
CL2	0.53	8.4	14.2
CL3	0.66	8.5	14.1
CL4	0.74	8.8	14.3
CL5	1.01	8.7	14
CL6	1.44	8.6	13.8
Mean ± Std	0.76 ± 0.38	8.60 ± 0.14	14.15 ± 0.22

**Table 2:** Description of the contact area and its relationship with Young’s modulus. The analysis is based on varying Young’s Modulus (E) but CL1 type base curve and diameter.

Young’s Modulus (E) (MPa)	Contact Area (mm^2^)
0.199	106.74
0.53	65.66
0.66	54.47
0.74	47.69
1.01	35.18
1.44	19.39

**Table 3:** Description of the contact area and its relationship with the CL base curve. The analysis is based on varying base curve values but CL1 type base curve and diameter.

Base Curve (Bc) (mm)	Contact Area (mm^2^)
8.4	127.96
8.5	118.55
8.6	117.31
8.7	111.27
8.8	106.74

**Table 4:** Description of the contact area and its relationship with CL diameter. The analysis is based on varying diameter values but CL1 type Young’s modulus and base curve.

Diameter (d3) (mm)	Contact Area (mm^2^)
13.8	93.07
14.0	95.45
14.1	99.59
14.2	102.07
14.3	105.01
14.5	106.74

**Table 5:** Correlation analysis between CL parameters (Young’s modulus, base curve, and diameter) and maximum displacements in X, Y, and Z directions, minimum stress and contact area, presented with 95% confidence intervals. Metrics include the Pearson correlation coefficient (r) and the *p*-value (*p*). * Represents a statistically significant correlation (*p* < 0.05). Calculations were performed with MATLAB toolbox [26].

CL Parameters	Maximum X Displacement		Maximum Y Displacement		Maximum Z Displacement		Minimum σ3 Stress		Contact Area	
	r	*p*	r	*p*	r	*p*	r	*p*	r	*p*
Young’s Modulus	0.941	0.005 *	0.935	0.006 *	0.995	4.3 × 10^−5^ *	−0.928	0.008 *	−0.947	0.004 *
Base curve	−0.444	0.454	−0.705	0.184	−0.572	0.313	−0.949	0.014 *	−0.977	0.004 *
Diameter	−0.660	0.154	−0.977	8.1 × 10^−4^ *	0.549	0.259	−0.836	0.038 *	0.976	8.4 × 10^−4^ *

## Data Availability

The original contributions presented in this study are included in the article; further inquiries can be directed to the corresponding author.

## Supplementary Materials

The following supporting information can be downloaded at: https://www.mdpi.com/article/10.3390/bioengineering11100966/s1, Section S1: Displacements in X, Y, Z directions for the back surface contact lens and outer cornea surface; Section S2: Comparison between Maximum Shear Stress and σ3; Section S3: Comparison of σ3 on back surface contact lens and the outer surface of the cornea.
