# Supplementary material for: Influence of Contact Lens Parameters on Cornea: Biomechanical Analysis
Source: Bioengineering (Basel). 2024 Sep 27;11(10):966. doi: 10.3390/bioengineering11100966 (PMC11505363; doi:10.3390/bioengineering11100966)
Supplement: Supplementary file 1 [file bioengineering-11-00966-s001.zip › bioengineering-3177139-supplementary.pdf]

INFLUENCE OF CONTACT LENS PARAMETERS ON CORNEA: BIOMECHANICAL ANALYSIS.

Supplementary Material

Darshan Ramasubramanian (D.R.)[1,2], José Luis Hernández Verdejo (J.L.)[1] and José Manuel López-Alonso (J.M.) [*,1].

[1] Faculty of Optics and Optometry, Complutense University of Madrid., Arcos de Jalón 118,28037, Madrid, MADRID, Spain.
[2] Alain Afflelou Óptico Portugal, Av. António Augusto de Aguiar, 11, 1050-016, Lisbon, Portugal.
[*] Correspondence: jmlopez@ucm.es; Tel.: +34 913946874.

Section S1: Displacements in X, Y, Z directions for the back surface contact lens and outer cornea surface.

Images for variations in all three contact lens parameters (Young module, base curve and lens diameter) have been included regarding the deformations of the back surface of the contact lens and the outer surface of the cornea. Additional images have been provided regarding the displacement in the XY plane, which focuses on the lateral movement as it is crucial for understanding the folding and twisting of the contact lens on the cornea. Displacement magnitude is also shown here, which provides a broader perspective of the total movement of the contact lens over the cornea. It is important to note that the front surface of the contact lens and the outer surface of the cornea are shown from the top view projection, while the back surface of the contact lens is viewed from the bottom view projection. As a result, the prism ballast on the back surface appears at the top due to the reversed axis rotation (refer to axis directions in the figures for clarification).

Figures S1, S2, and S3 show the deformations of the back surface of the contact lens when varying Young's modulus, base curve, and diameter, respectively. Each row in Figure S1 corresponds to different Young's modulus values, each row in Figure S2 represents base curve variations, and Figure S3 corresponds to diameter variations. The columns in all three figures are structured similarly: columns 1, 2, and 3 represent X, Y, and Z displacements, column 4 illustrates displacement in the XY plane, and column 5 shows displacement magnitude. In this study, the back surface of the contact lens exhibits deformation patterns similar to those of the front surface, which is why we chose not to include these results. The maximum and minimum displacement values are also close to those on the front surface. Since the front surface with prism ballast provides a more detailed understanding of the contact lens behaviour, it was prioritised over the back surface.

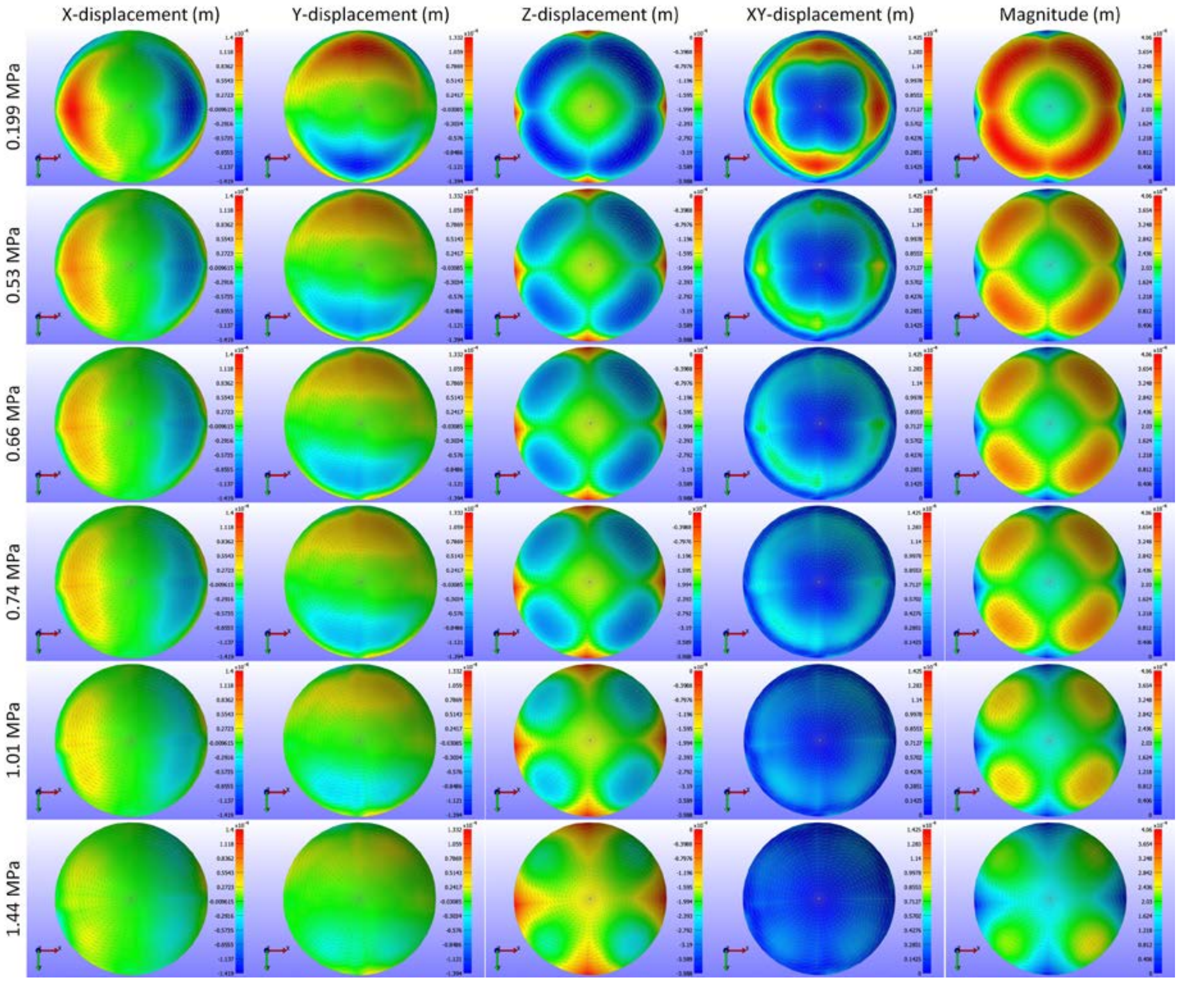

Figure S1: Deformation of the back surface of the contact lens with varying Young's modulus. Each row corresponds to a different Young's modulus value. Columns 1, 2, and 3 represent the X, Y, and Z displacements, column 4 illustrates the displacement in the XY plane, and column 5 shows the displacement magnitude.

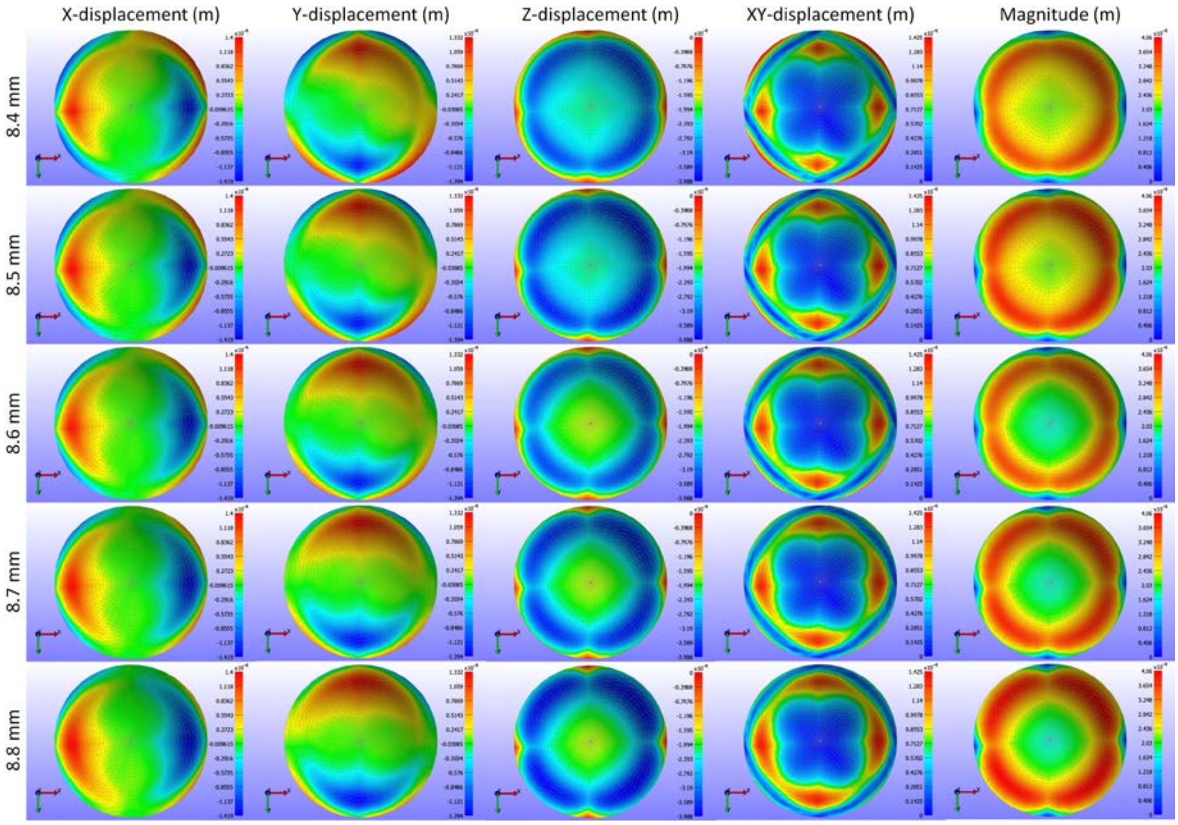

Figure S2: Deformation of the back surface of the contact lens with varying base curves. Each row corresponds to a different base curve value. Columns 1, 2, and 3 represent the X, Y, and Z displacements, column 4 illustrates the displacement in the XY plane, and column 5 shows the displacement magnitude.

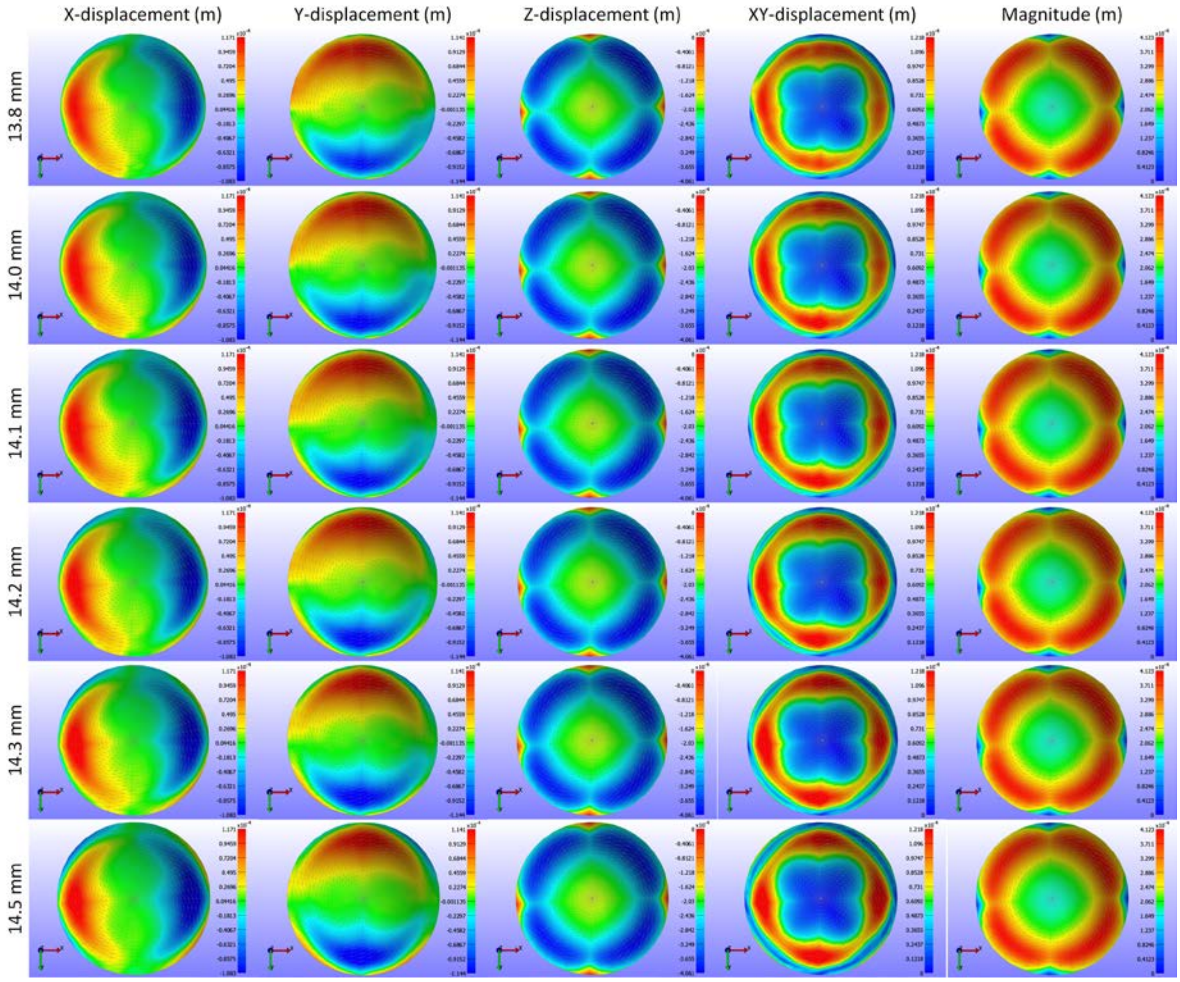

Figure S3: Deformation of the back surface of the contact lens with varying contact lens diameter. Each row corresponds to a different diameter value. Columns 1, 2, and 3 represent the X, Y, and Z displacements, column 4 illustrates the displacement in the XY plane, and column 5 shows the displacement magnitude.

Figures S4, S5, and S6 illustrate the deformation of the outer surface of the cornea, also varying by Young's modulus, base curve, and diameter. Each row in Figure S1 corresponds to different Young's modulus values, each row in Figure S2 represents base curve variations, and Figure S3 corresponds to diameter variations. The first two columns show the isometric view of the contact lens before and after fitting. Columns 3, 4, and 5 represent the X, Y, and Z displacements, column 6 illustrates the displacement in the XY plane, and column 7 shows the displacement magnitude. As shown in the images, the deformation of the cornea is in microns, indicating that it is relatively small. While the contact lens movement does affect the cornea, the displacements in all directions are much smaller on the cornea than on the lens. For the lens back surface, the magnitude of the displacement in XY direction is the biggest in the final stage of movement due to the spreading of this back lens surface over the cornea.

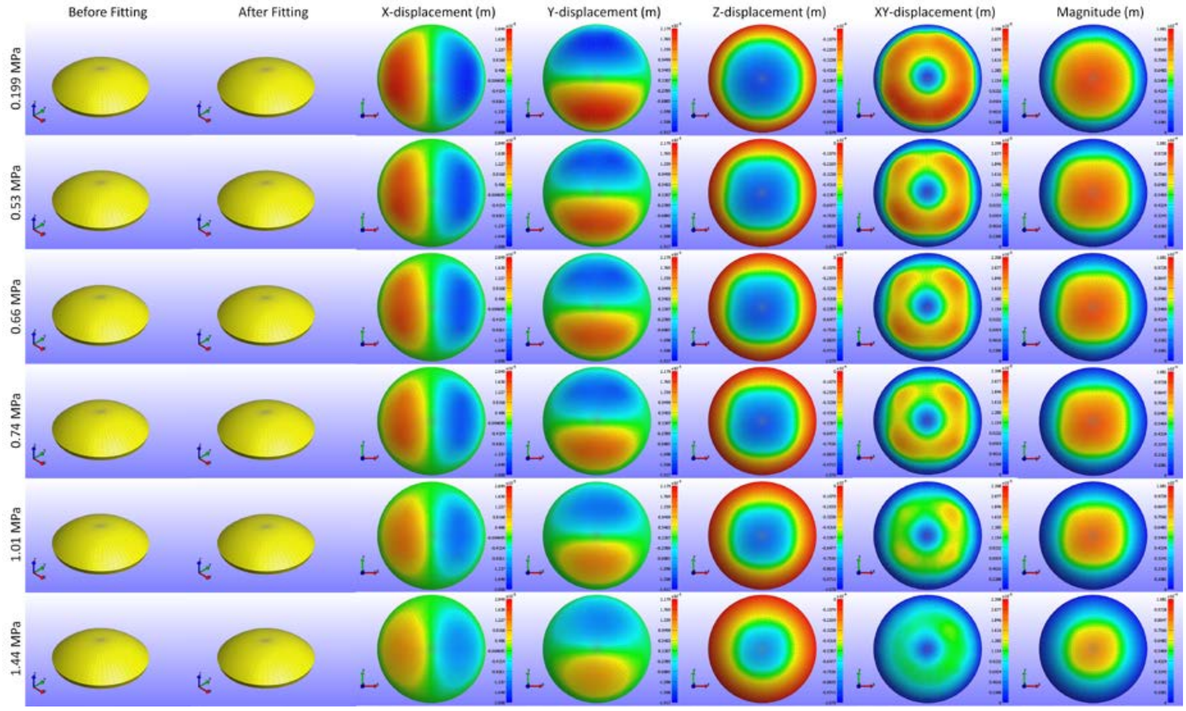

Figure S4: Deformation of the outer surface of the cornea with varying Young's modulus. Each row corresponds to a different Young's modulus value. The first two columns show the isometric view of the cornea before and after fitting. Columns 3, 4, and 5 represent the X, Y, and Z displacements, column 6 illustrates the displacement in the XY plane, and column 7 shows the displacement magnitude.

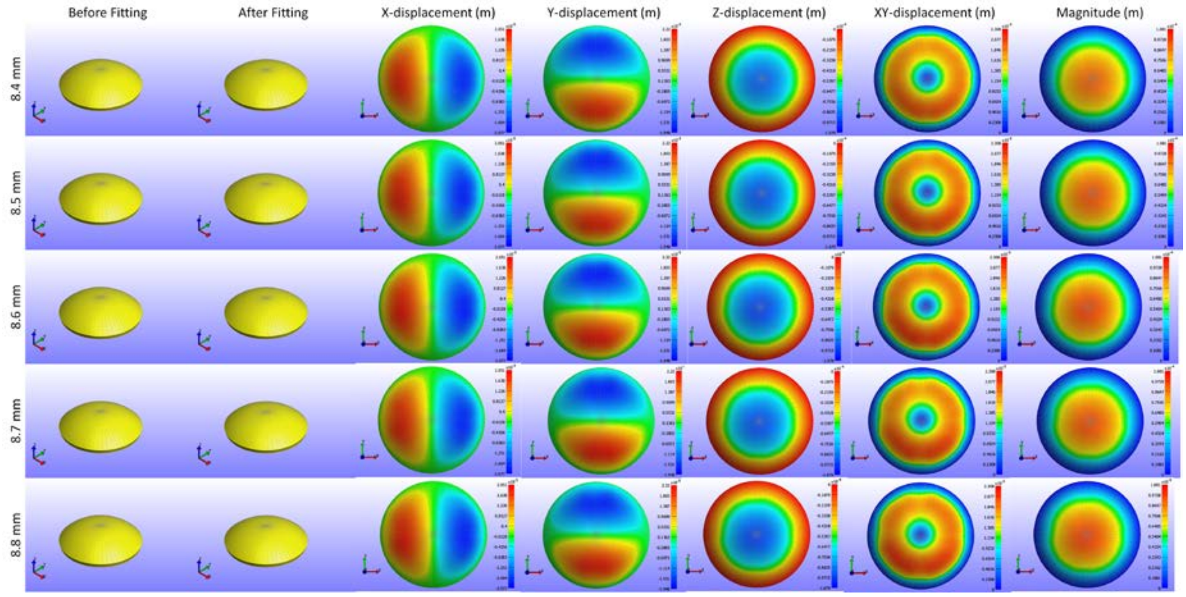

Figure S5: Deformation of the outer surface of the cornea with varying base curves. Each row corresponds to a different base curve value. The first two columns show the isometric view of the cornea before and after fitting. Columns 3, 4, and 5 represent the X, Y, and Z displacements, column 6 illustrates the displacement in the XY plane, and column 7 shows the displacement magnitude.

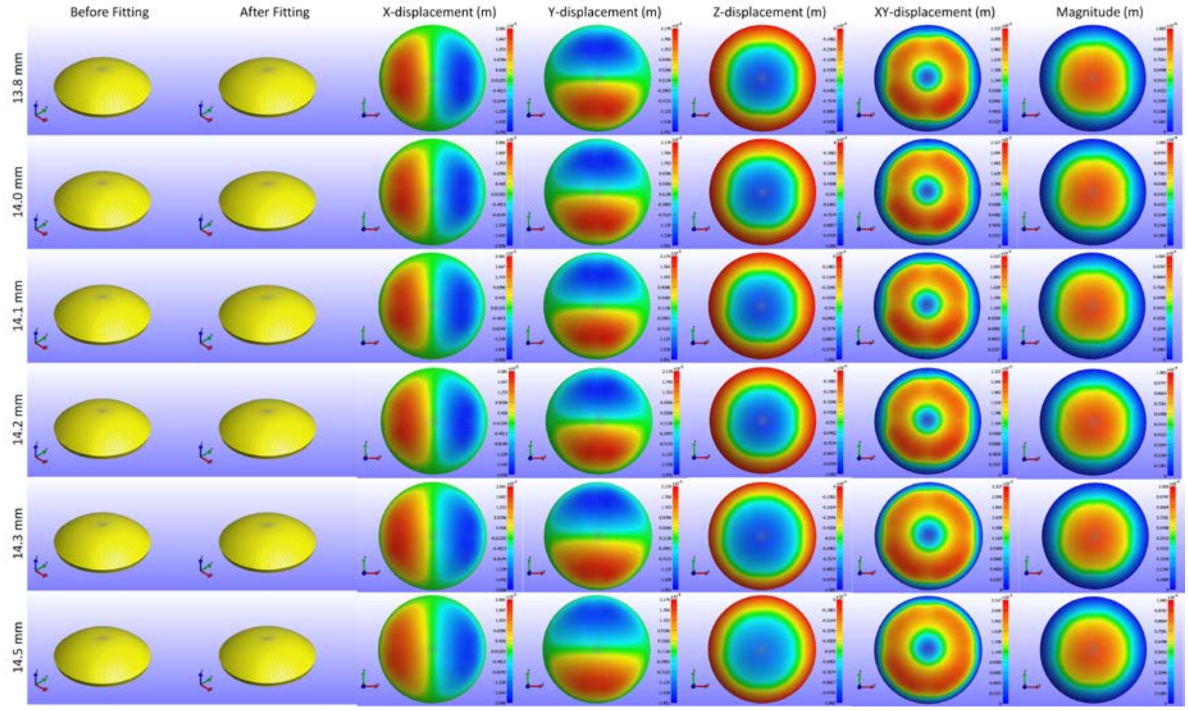

Figure S6: Deformation of the outer surface of the cornea with varying contact lens diameter. Each row corresponds to a different diameter value. The first two columns show the isometric view of the cornea before and after fitting. Columns 3, 4, and 5 represent the X, Y, and Z displacements, column 6 illustrates the displacement in the XY plane, and column 7 shows the displacement magnitude.

Section S2: Comparison between Maximum Shear Stress and σ3

In FEBio, the maximum shear stress $\Xi(F)$ is defined using all three principal stresses σ1, σ2, σ3, as shown in the equation below (equation 1). This formula calculates the shear stress between each pair of principal stresses and selects the highest value, ensuring that the most critical shear stress condition is captured. This approach is based on the theory that predicts material failure when the absolute maximum shear stress reaches the yield stress observed in a simple tension test and is often used in the case of ductile materials such as copper, gold, etc. or brittle materials such as concrete, ceramics, etc.

$$\Xi(F) = \max(\frac{|\sigma_1 - \sigma_2|}{2}, \frac{|\sigma_2 - \sigma_3|}{2}, \frac{|\sigma_3 - \sigma_1|}{2}) \quad (1)$$

Figures S7 through S15 present the maximum shear stress and the three principal stresses on the front surface of the contact lens, the back surface of the contact lens, and the cornea. Figures S7 through S9 illustrate variations in Young's modulus on the front surface of the contact lens, the back surface of the contact lens and the cornea, respectively, with each row representing different Young's modulus values. Figures S10 through S12 show variations in base curve values on the front surface of the contact lens, the back surface of the contact lens, and the cornea, respectively, with each row corresponding to a different base curve. Lastly, Figures S13 through S15 depict variations in contact lens diameter on the front surface of the contact lens, the back surface of the contact lens and the cornea, respectively, where each row represents a different diameter. The first column in these figures shows the 3-principal stress, while the second column displays the maximum shear stress. It is important to note that the front surface of the contact lens and the outer surface of the cornea are shown from the top

view, while the back surface of the contact lens is viewed from the bottom. As a result, the prism ballast on the back surface appears at the top due to the reversed axis rotation (refer to axis directions in the figures for clarification).

The figures show that the maximum shear stress and the 3-principal stress maps exhibit similar distribution patterns across the surface. The value of both magnitudes differs, being the modulus of 3-principal stress larger in magnitude than the maximum shear stress (the absolute value is taken because maximum shear stress is defined as an absolute value too). The maximum shear stress is defined as an absolute value of differences between different components of 3-principal stress. So, in general, its magnitude could be lower than one particular component.

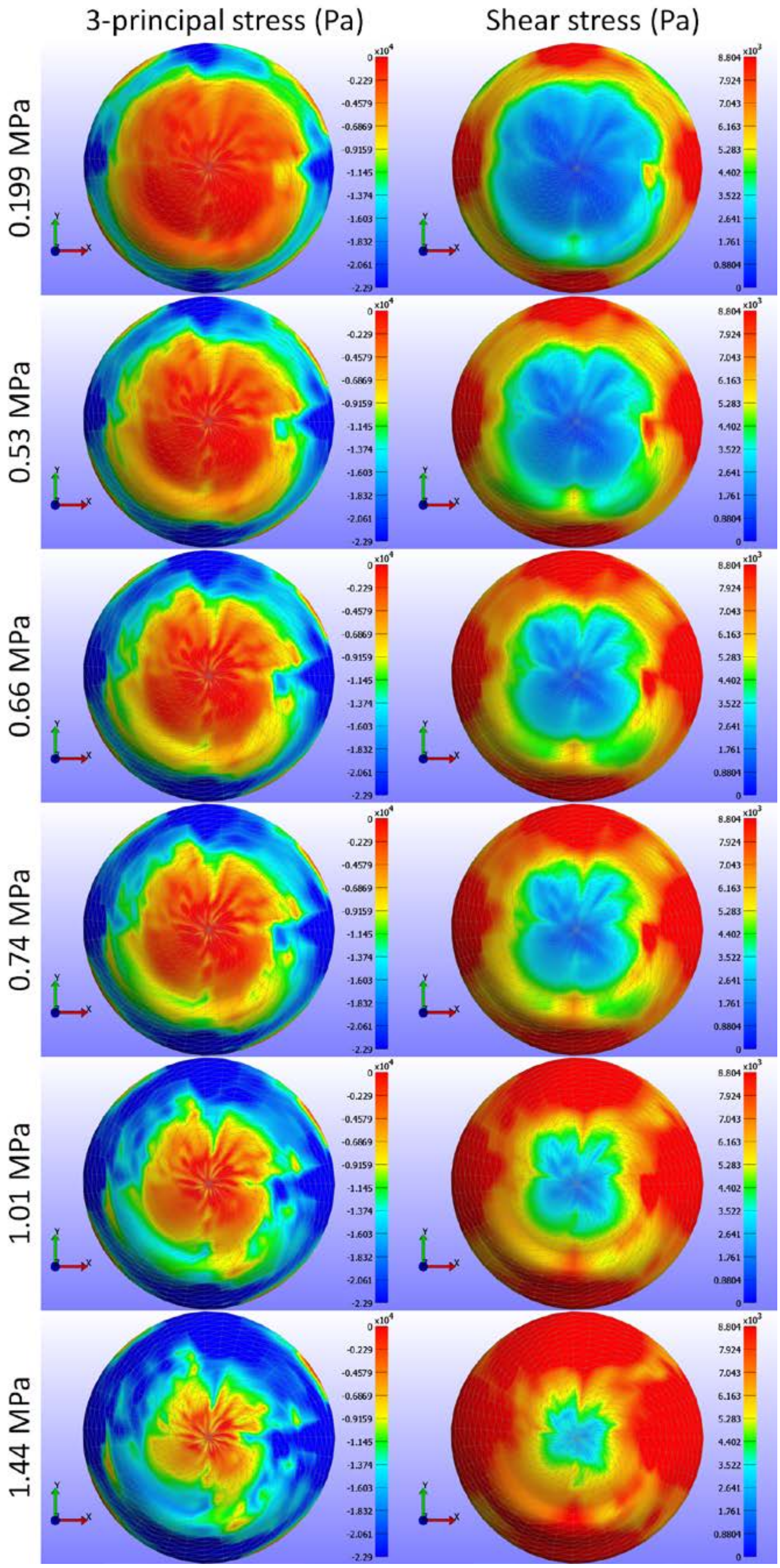

Figure S7: Stress maps on the front surface of the contact lens with varying Young's modulus. Each row corresponds to a different Young's modulus value. Column 1 represents the 3-principal stress (σ3), and column 2 shows the maximum shear stress map.

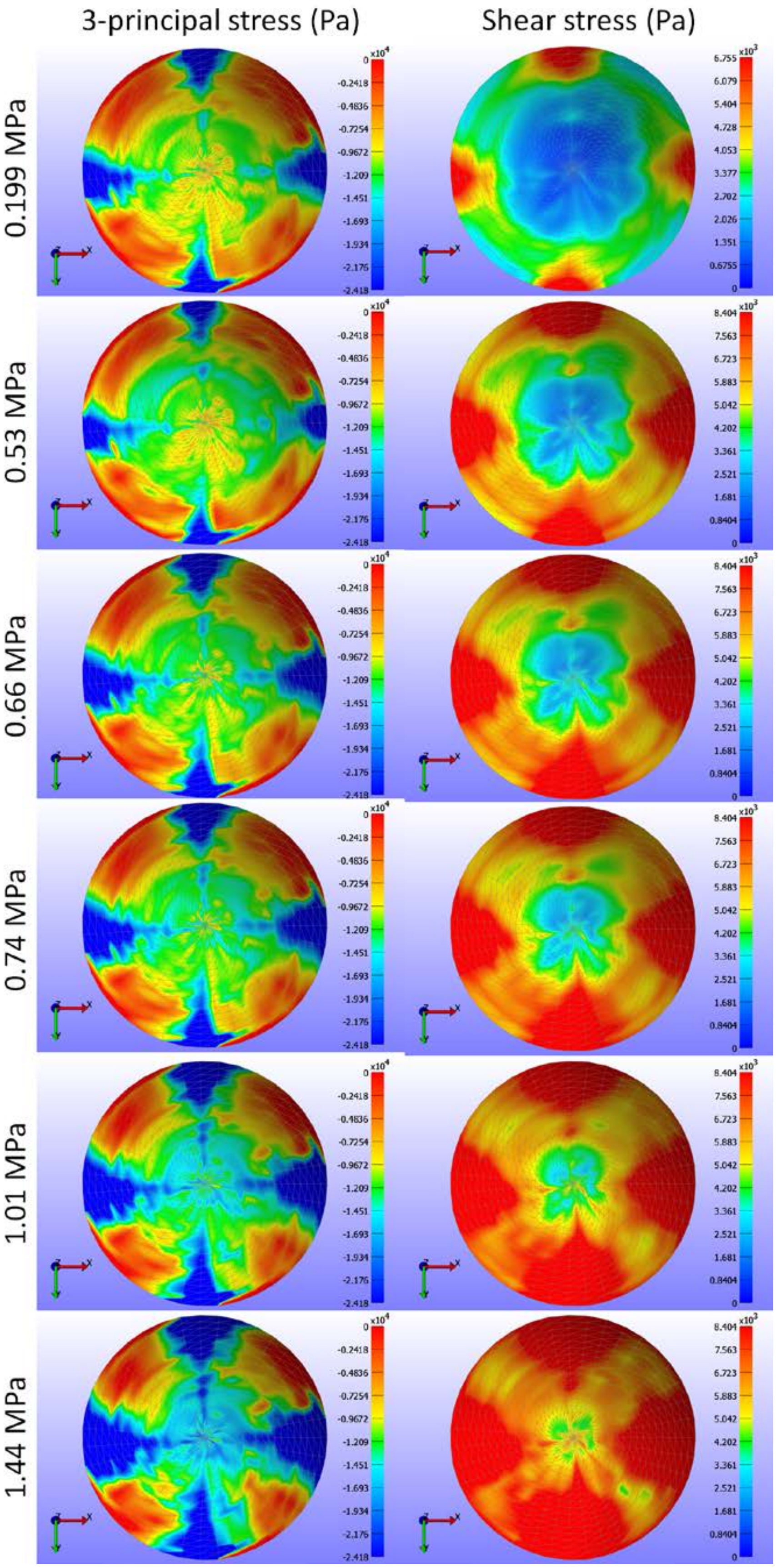

Figure S8: Stress maps on the back surface of the contact lens with varying Young's modulus. Each row corresponds to a different Young's modulus value. Column 1 represents the 3-principal stress (σ3), and column 2 shows the maximum shear stress map.

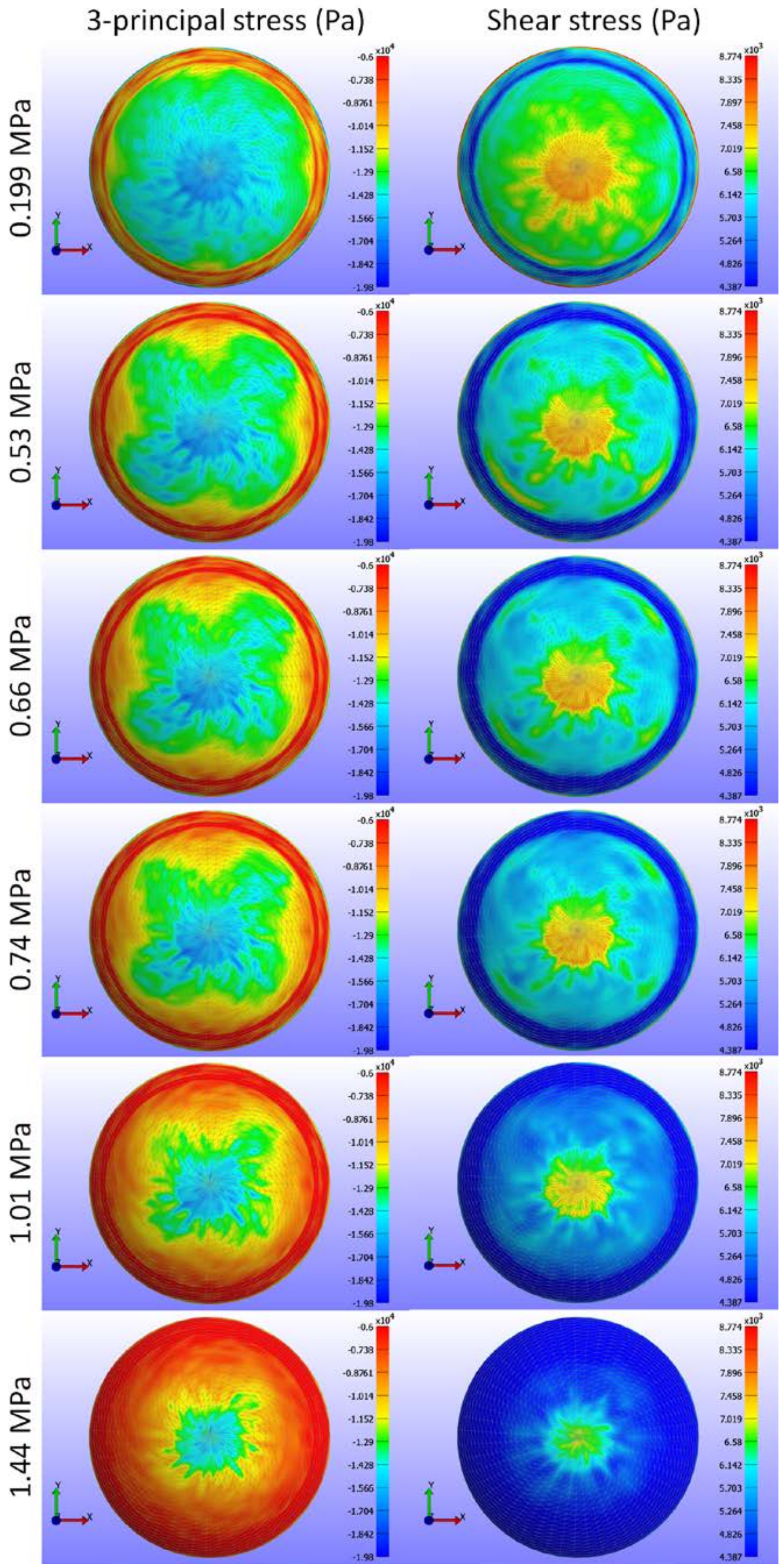

Figure S9: Stress maps on the outer surface of the cornea with varying Young’s modulus. Each row corresponds to a different Young’s modulus value. Column 1 represents the 3-principal stress (σ3), and column 2 shows the maximum shear stress map.

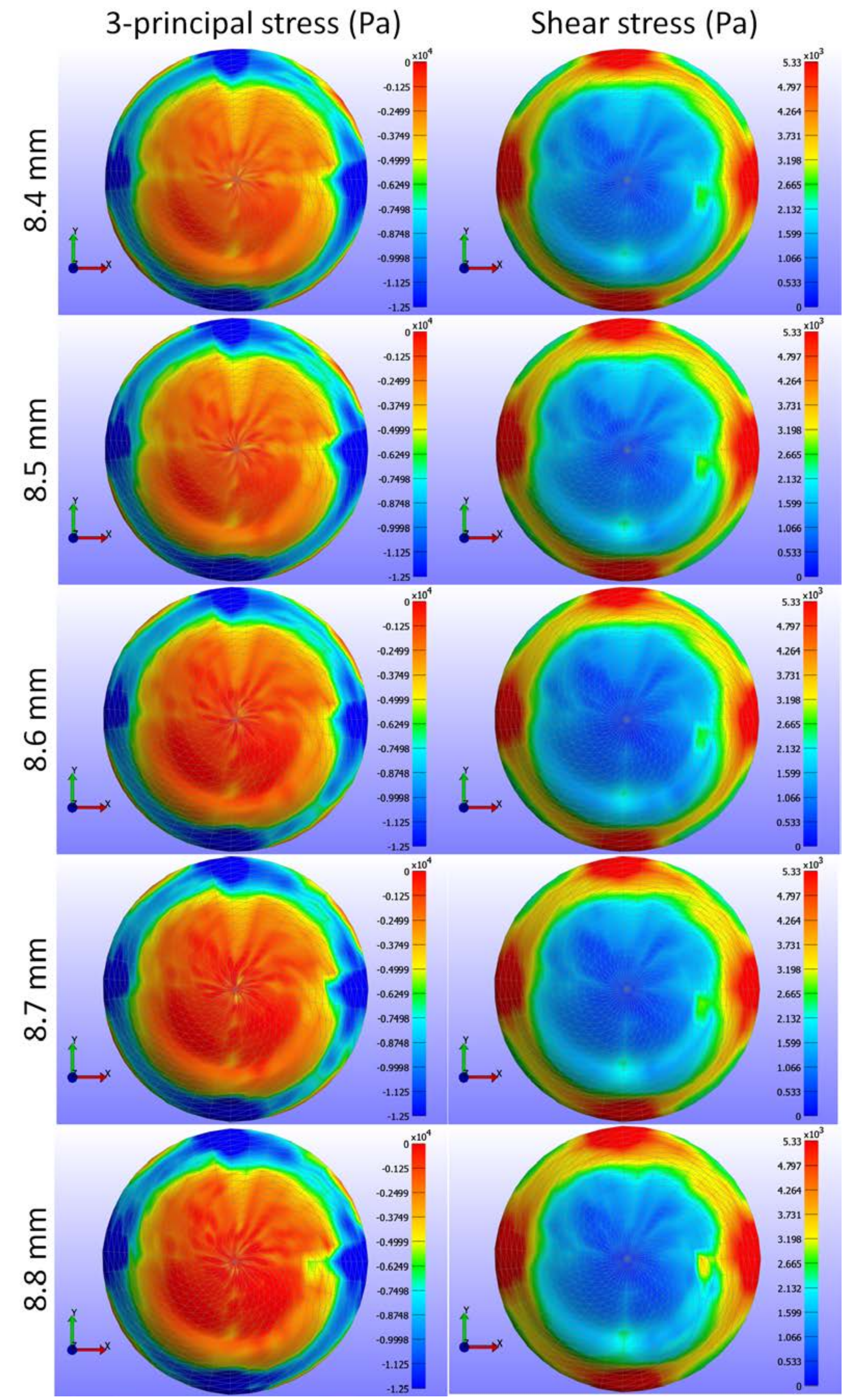

Figure S10: Stress maps on the front surface of the contact lens with varying base curves. Each row corresponds to a different base curve value. Column 1 represents the 3-principal stress (σ3), and column 2 shows the maximum shear stress map.

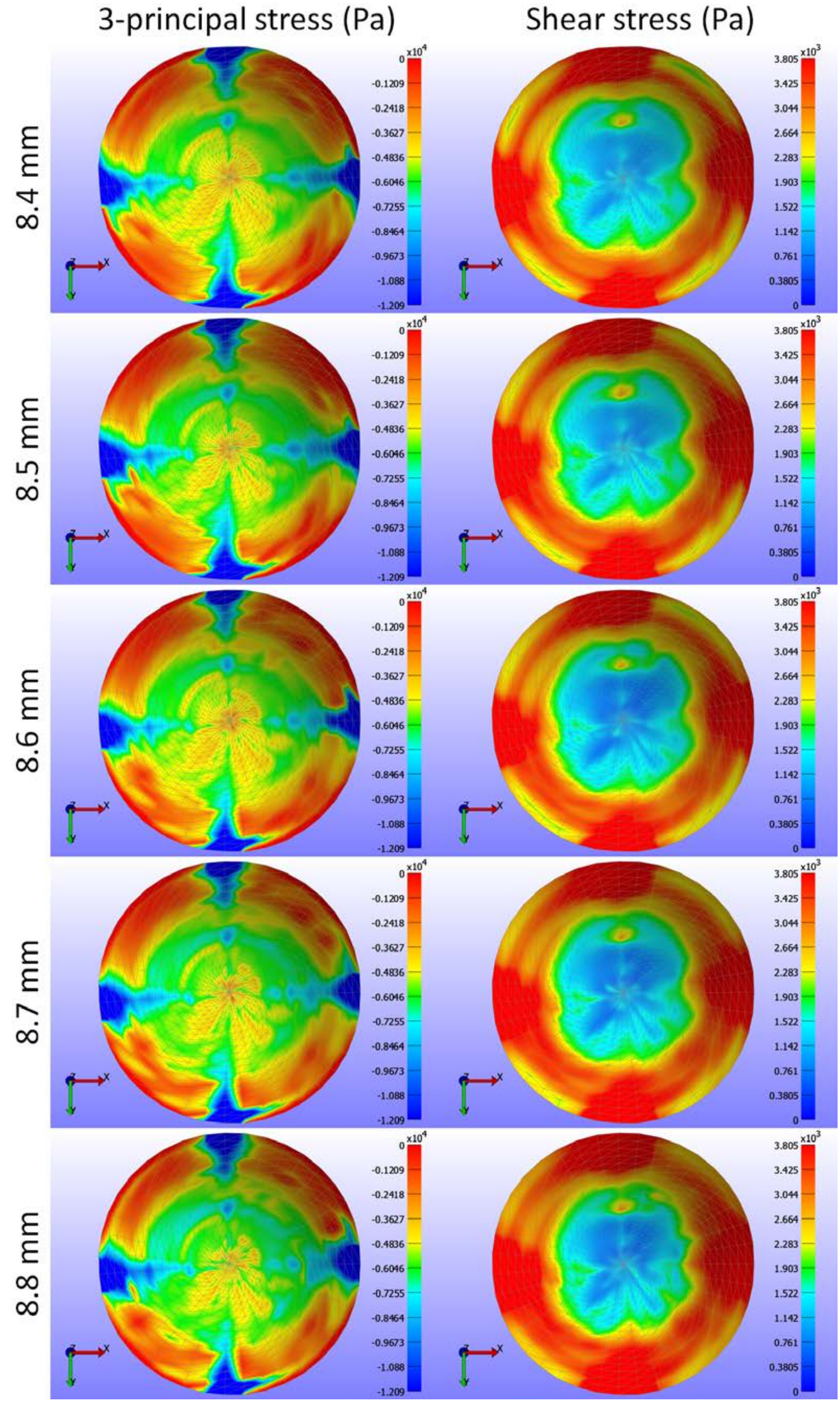

Figure S11: Stress maps on the back surface of the contact lens with varying base curves. Each row corresponds to a different base curve value. Column 1 represents the 3-principal stress (σ3), and column 2 shows the maximum shear stress map.

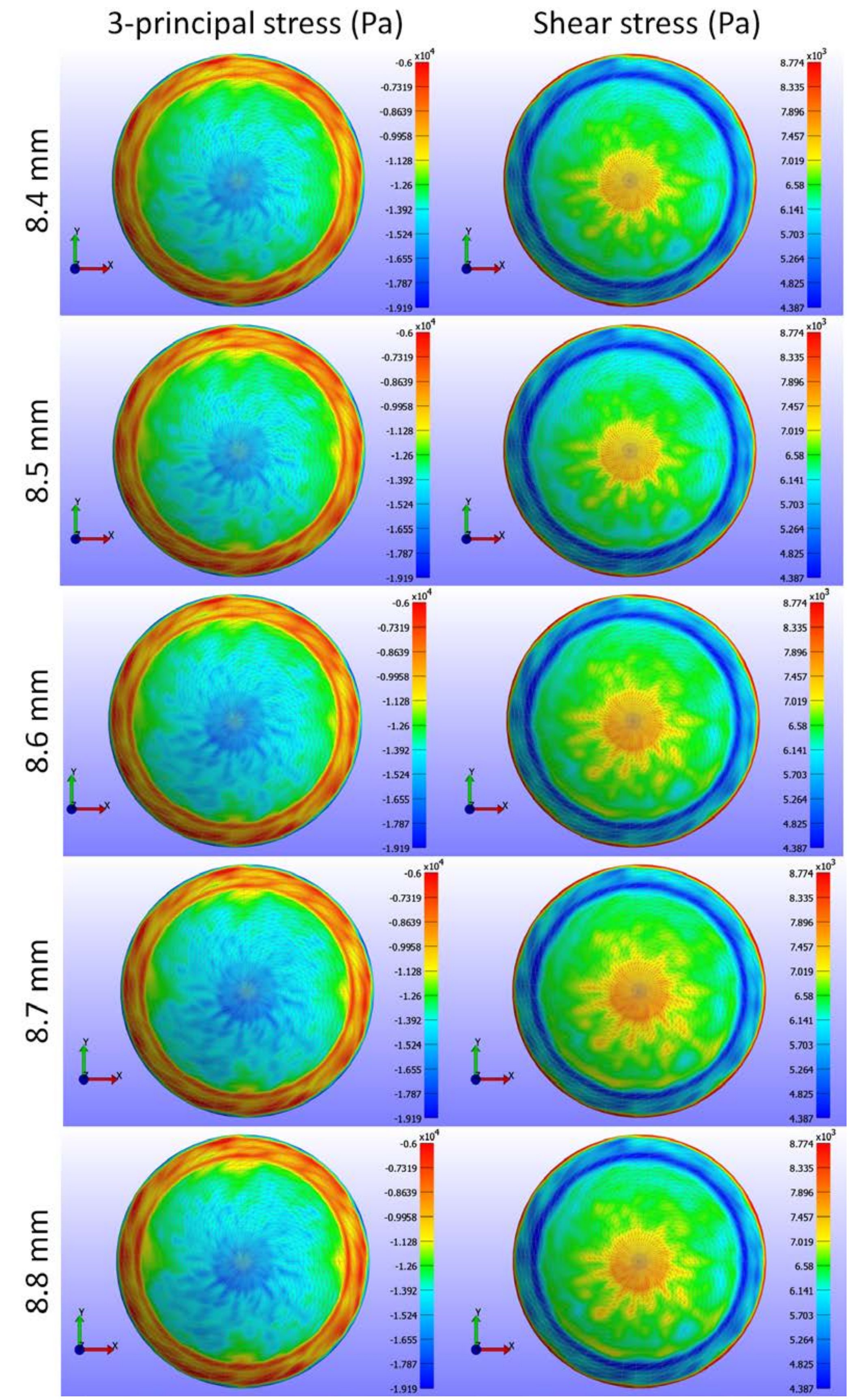

Figure S12: Stress maps on the outer surface of the cornea with varying base curves. Each row corresponds to a different base curve value. Column 1 represents the 3-principal stress (σ3), and column 2 shows the maximum shear stress map.

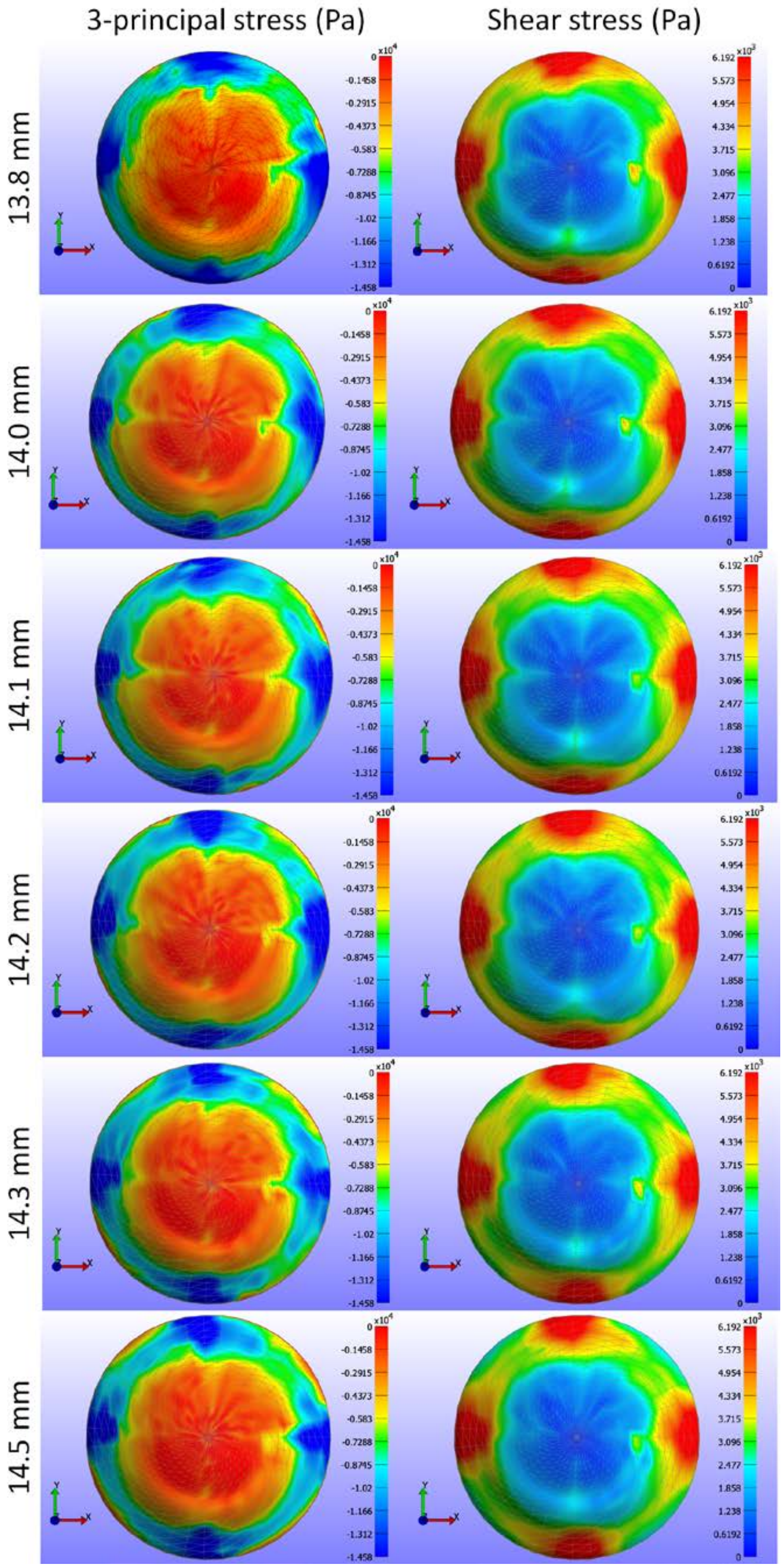

Figure S13: Stress maps on the front surface of the contact lens with varying contact lens diameters. Each row corresponds to a different diameter value. Column 1 represents the 3-principal stress (σ3), and column 2 shows the maximum shear stress map.

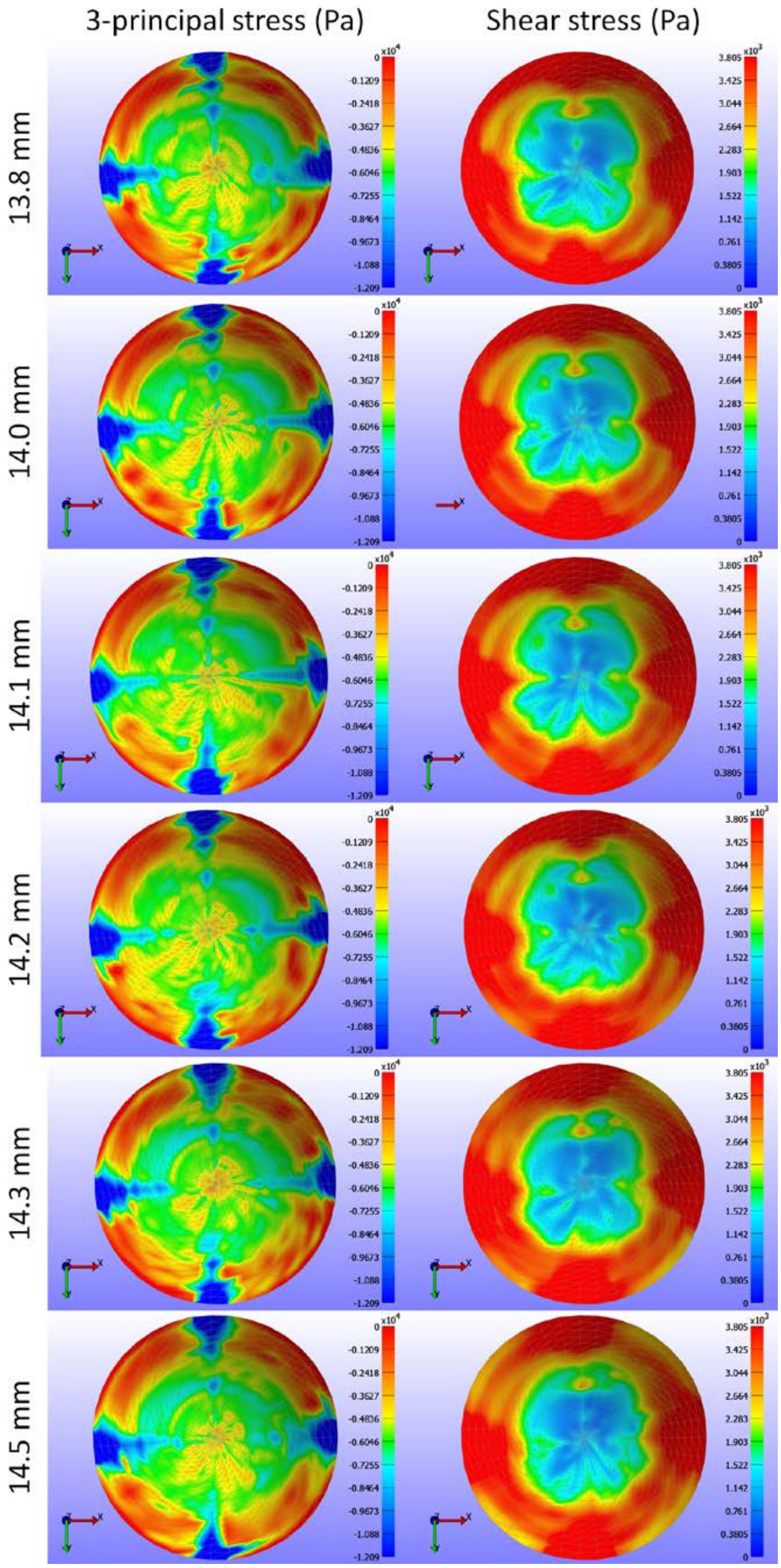

Figure S14: Stress maps on the back surface of the contact lens with varying contact lens diameters. Each row corresponds to a different diameter value. Column 1 represents the 3-principal stress (σ3), and column 2 shows the maximum shear stress map.

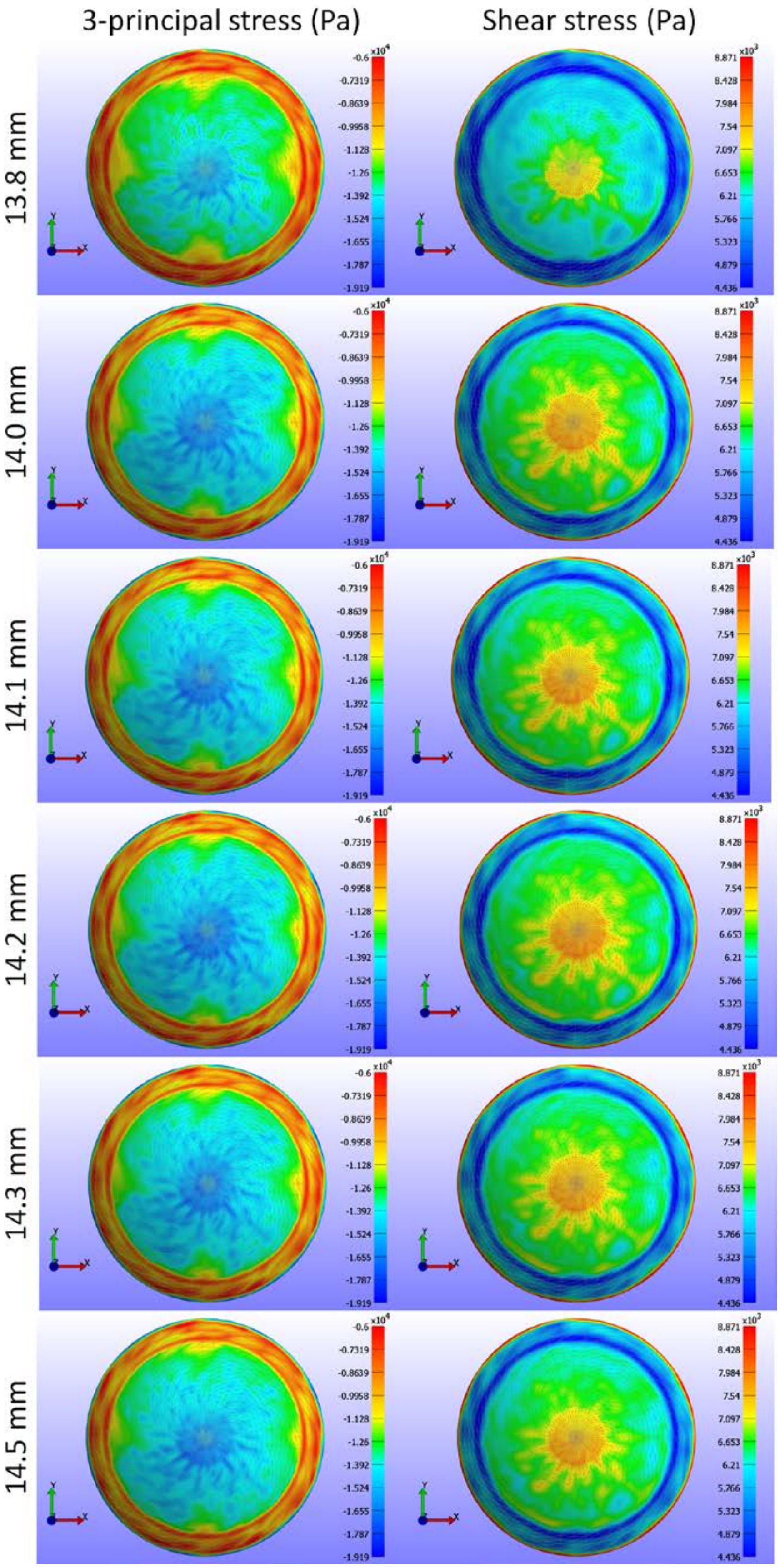

Figure S15: Stress maps on the outer surface of the cornea with varying contact lens diameters. Each row corresponds to a different diameter value. Column 1 represents the 3-principal stress (σ3), and column 2 shows the maximum shear stress map.

In order to further analyse the relationship between the magnitudes of both quantities, a correlation analysis and linear adjustment have been carried out for both. For each type of parameter studied (Young modulus, base curve and diameter), the maximum, mean and minimum variation values have been taken. For each of these simulations, the relationship between the modulus of the value of the 3-principal stress and the maximum shear stress in the cornea, the anterior and posterior surfaces of the contact lens has been studied. Representing, for each of these surfaces, the modulus of the 3-principal stress on the x-axis and the maximum shear stress on the y-axis, the regression line y=p1x+p2 is obtained, giving the values of p1 p2 with their 95% confidence intervals, the R2 of the adjustment and the Pearson correlation between the two variables (see figure S16 for an example). All these calculations have been carried out using routines implemented in MATLAB. For each of these values, an estimate of the standard deviation is also obtained by means of the three simulations that are taken for each type of parameter (Young modulus, base curve and diameter). The results are presented in the tables S1; S2 and S3.

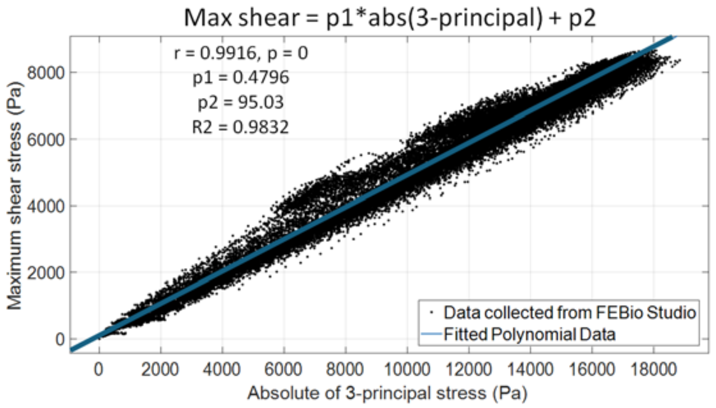

Figure S16: Linear fitting of the relationship between Maximum shear stress and the absolute value of the 3-principal stress on the outer surface of the cornea for the simulation of the Young's modulus = 0.199 MPa, Base curve = 8.6 mm and Diameter = 14.5 mm. Parameters r,p represents the Pearson correlation coefficient and p-value.

Table S1: Relation between Maximum Shear Stress and 3-principal stress on cornea. Pearson correlation coefficient and p-value (r, p). Values of linear fitting for Maximun Shear stress vs. absolute value of 3-principal stress (Max. Shear=p1*abs(3-principal)+p2, and R^2 of the fitting. The ± is the standard deviation through the simulations taken for each variation parameter.

<table>
<tr><th rowspan="2">CL parameters</th><th colspan="2">Correlation analysis</th><th colspan="2">p1</th><th colspan="2">p2</th><th rowspan="2">R-square</th></tr>
<tr><th>r</th><th>p</th><th>Lower bound</th><th>Upper bound</th><th>Lower bound</th><th>Upper bound</th></tr>
<tr><td rowspan="2">Young's modulus</td><td rowspan="2">0.977 ± 0.0106</td><td rowspan="2">0</td><td colspan="2">0.464 ± 0.002</td><td colspan="2">345.07 ± 54.28</td><td rowspan="2">0.954 ± 0.011</td></tr>
<tr><td>0.463 ± 0.003</td><td>0.466 ± 0.003</td><td>334.47 ± 52.76</td><td>355.66 ± 55.80</td></tr>
<tr><td rowspan="2">Base curve</td><td rowspan="2">0.984 ± 0.012</td><td rowspan="2">0</td><td colspan="2">0.475 ± 0.009</td><td colspan="2">199.65 ± 175.56</td><td rowspan="2">0.969 ± 0.023</td></tr>
<tr><td>0.474 ± 0.009</td><td>0.476 ± 0.009</td><td>190.72 ± 172.90</td><td>208.92 ± 177.92</td></tr>
<tr><td rowspan="2">Diameter</td><td rowspan="2">0.982 ± 0.011</td><td rowspan="2">0</td><td colspan="2">0.471 ± 0.006</td><td colspan="2">238.48 ± 141.96</td><td rowspan="2">0.965 ± 0.019</td></tr>
<tr><td>0.470 ± 0.006</td><td>0.472 ± 0.005</td><td>228.69 ± 140.05</td><td>248.27 ± 143.87</td></tr>
</table>

Table S2: Relation between Maximum Shear Stress and 3-principal stress on front surface of the contact lens. Pearson correlation coefficient and p-value (r, p). Values of linear fitting for Maximun Shear stress vs. absolute value of 3-principal stress (Max. Shear=p1*abs(3-principal)+p2, and R^2 of the fitting. The ± is the standard deviation through the simulations taken for each variation parameter.

**CL parameters**	**Correlation analysis**		**p1**		**p2**		**R-square**
	**r**	**p**	**Lower bound**	**Upper bound**	**Lower bound**	**Upper bound**	
Young's modulus	0.911 ± 0.022	0	0.435 ± 0.038		805.85 ± 228.77		0.829 ± 0.039
			0.433 ± 0.038	0.438 ± 0.039	780.42 ± 226.23	828.39 ± 247.65	
Base curve	0.944 ± 0.010	0	0.404 ± 0.008		495.28 ± 49.97		0.877 ± 0.008
			0.402 ± 0.008	0.406 ± 0.008	482.55 ± 40.60	501.30 ± 39.72	
Diameter	0.936 ± 0.004	0	0.389 ± 0.006		476.18 ± 74.84		0.965 ± 0.019
			0.388 ± 0.005	0.391 ± 0.006	465.00 ± 69.30	480.65 ± 70.98	

Table S3: Relation between Maximum Shear Stress and 3-principal stress on the back surface ot the contact lens. Pearson correlation coefficient and p-value (r, p). Values of linear fitting for Maximun Shear stress vs. absolute value of 3-principal stress (Max. Shear=p1*abs(3-principal)+p2, and R^2 of the fitting. The ± is the standard deviation through the simulations taken for each variation parameter.

**CL parameters**	**Correlation analysis**		**p1**		**p2**		**R-square**
	**r**	**p**	**Lower bound**	**Upper bound**	**Lower bound**	**Upper bound**	
Young's modulus	0.724 ± 0.158	0	0.308 ± 0.047		1291.10 ± 545.04		0.541 ± 0.022
			0.305 ± 0.048	0.312 ± 0.046	1256.7 ± 536.12	1325.5 ± 553.98	
Base curve	0.506 ± 0.044	0	0.252 ± 0.019		700.24 ± 34.80		0.257 ± 0.045
			0.245 ± 0.019	0.256 ± 0.019	675.99 ± 34.87	724. 49 ± 34.75	
Diameter	0.568 ± 0.018	0	0.266 ± 0.006		739.14 ± 78.69		0.323 ± 0.020
			0.261 ± 0.006	0.271 ± 0.006	580.22 ± 167.99	816.95 ± 186.97	

These results show a high correlation for all surfaces between the distribution of the Maximum Search Stress and the 3-principal stress, being much lower only in the case of the back surface of the contact lens. For this surface, these spatial distributions can be seen in figure 1.38 where the maximum values of the Maximum Shear Stress occur at the periphery of the lens with a circular symmetry, not so noticeable in the case of the 3-principal stress. For this surface the absolute value of the magnitude of the displacement is always greater in the XY plane than in Z (see figures S1 to S3). This means that the deformation of the lens on this back surface is carried out fundamentally in the XY plane and not so much in the Z axis as it occurs for the rest of the surfaces (this back surface has to deform more in the XY plane

as it adapts to the contour of the cornea). Hence the lower correlation between the Maximum Shear Stress and the 3-principal stress.

Section S3: Comparison of σ3 on back surface contact lens and outer surface of the cornea

Figures S17, S18 and S19 present sections of the three principal stresses, including the cornea and sclera. As shown in Figure S17, Young's modulus substantially influences the stress distribution, similar to the differences observed in the deformation patterns in Section 1 of the present Supplementary Material. This highlights the model's sensitivity to variations in Young's modulus, confirming that it plays a critical role in determining stress distribution and material behaviour.

In contrast, Figures S18 and S19 show that changes in the base curve and diameter have a minimal effect on the three principal stresses, indicating that their influence on the material's stress response is comparatively less significant. The stress maps remain relatively unchanged across variations in these parameters. This further supports the conclusion that Young's modulus is a critical factor in the material's mechanical response in this study, while base curve and diameter exert far less influence.

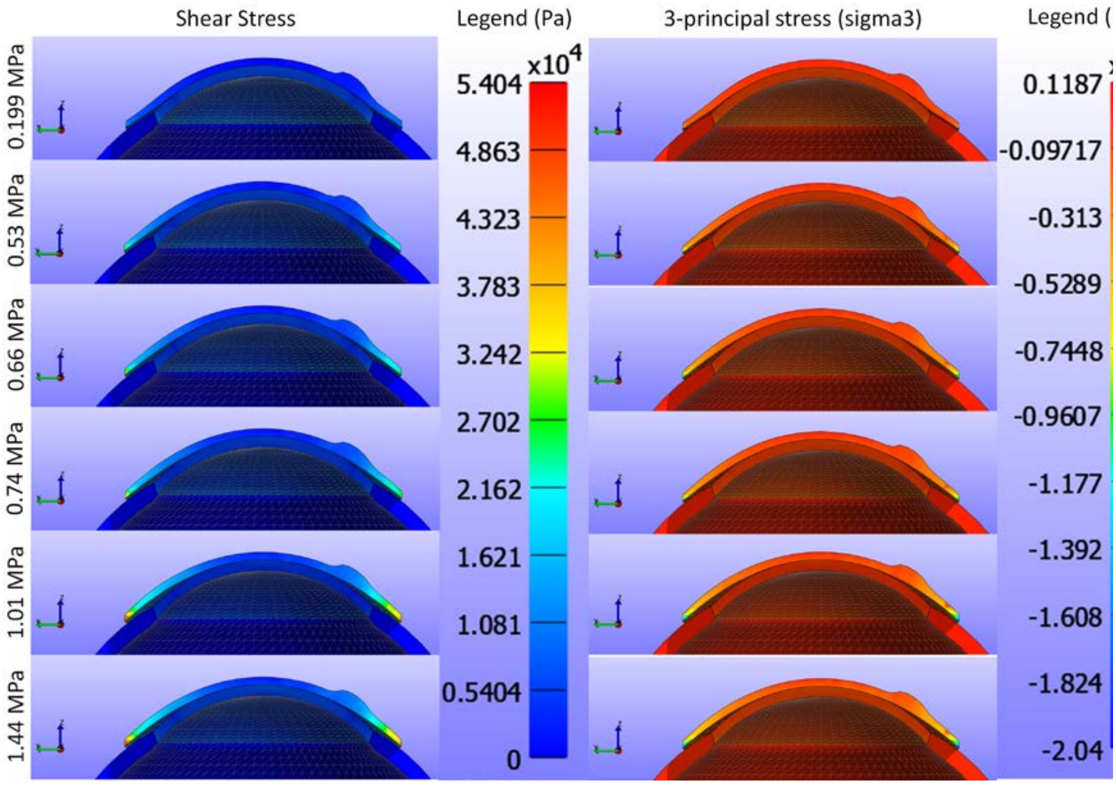

Figure S17: Cross-sectional stress maps of the model, including the cornea, sclera, and contact lens, with variations in Young's modulus.

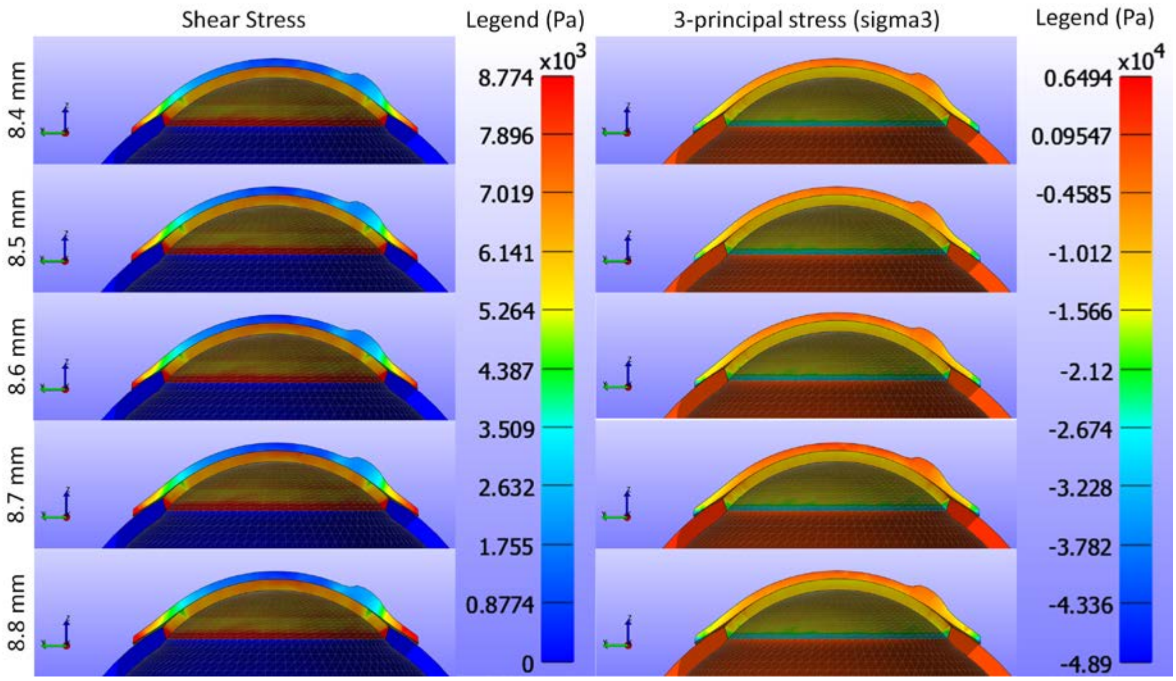

Figure S19: Cross-sectional stress maps of the model, including the cornea, sclera, and contact lens, with variations in the base curve.

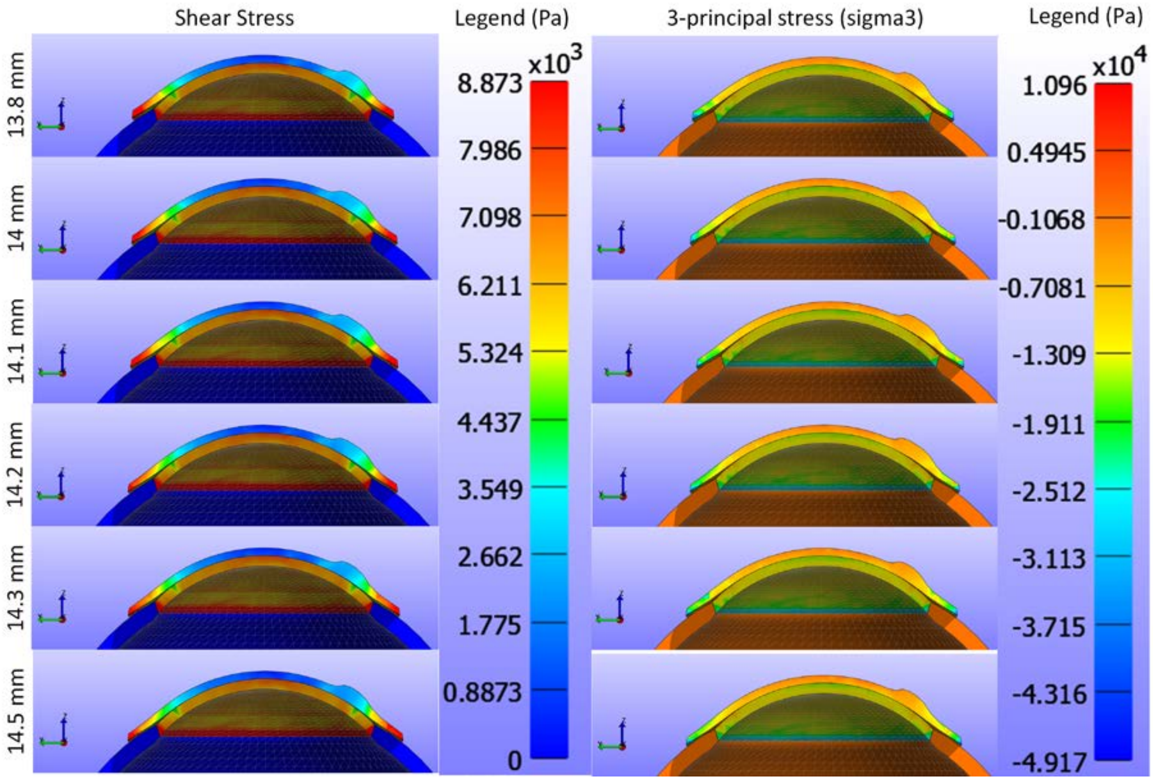

Figure S19: Cross-sectional stress maps of the model, including the cornea, sclera, and contact lens, with variations in contact lens diameter.

The 3-principal stress maps (σ3) for both the contact lens's back surface and the cornea's outer surface are represented in Figures S7 through S15. It is possible to see a more clear comparison in figure S20, where the 3-principal stress o contact lens’s back surface is plotted with the outer cornea 3-principal stress for some selected simulations. As noted in the previous Sections of the Supplementary Material, the main deformation of the posterior

surface of the lens occurs in the XY direction and not in Z, where it does occur for the case of the cornea, which is evidenced again by this comparison between the 3-principal stress for these two surfaces.

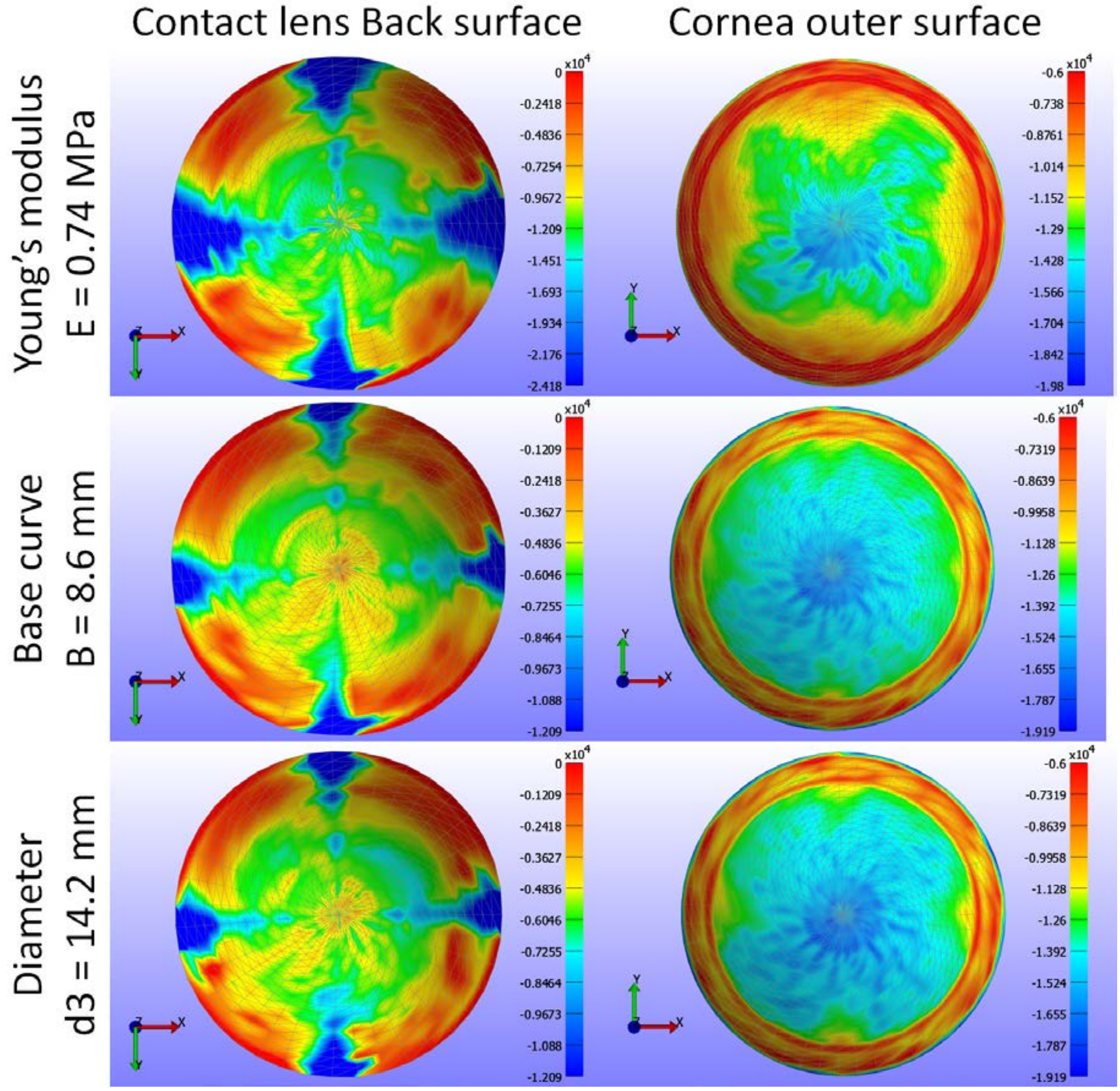

Figure S20. Comparison of 3-principal stress between the back surface of the contact lens and the outer surface of the cornea. Row 1 describes the stress maps for Young's modulus = 0.74 MPa, Base curve = 8.8 mm and Diameter = 14.5 mm. Row 2 describes the stress maps for Young's modulus = 0.199 MPa, Base curve = 8.6 mm and Diameter = 14.5 mm. Row 3 describes the stress maps for Young's modulus = 0.199 MPa, Base curve = 8.8 mm and Diameter = 14.2 mm. Column 1 represents the back surface of the contact lens and column 2 represents the outer surface of the cornea.
